# Mucosal bivalent live attenuated vaccine protects against human metapneumovirus and respiratory syncytial virus in mice

**DOI:** 10.1038/s41541-024-00899-9

**Published:** 2024-06-19

**Authors:** Daniela Ogonczyk-Makowska, Pauline Brun, Clémence Vacher, Caroline Chupin, Clément Droillard, Julie Carbonneau, Emilie Laurent, Victoria Dulière, Aurélien Traversier, Olivier Terrier, Thomas Julien, Marie Galloux, Stéphane Paul, Jean-François Eléouët, Julien Fouret, Marie-Eve Hamelin, Andrés Pizzorno, Guy Boivin, Manuel Rosa-Calatrava, Julia Dubois

**Affiliations:** 1 Centre de Recherche en Infectiologie of the Centre Hospitalier Universitaire de Québec and Université Laval, Québec, QC G1V 4G2 Canada; 2 International Research Laboratory RESPIVIR France - Canada, Centre de Recherche en Infectiologie, Faculté de Médecine RTH Laennec, 69008, Lyon, France, Université Claude Bernard Lyon 1, Université de Lyon, INSERM, CNRS, ENS de Lyon, France, Centre Hospitalier Universitaire de Québec - Université Laval, QC G1V 4G2, Québec, Canada; 3 CIRI, Centre International de Recherche en Infectiologie, Team VirPath, INSERM U1111, CNRS UMR 5308, ENS de Lyon, Université Claude Bernard Lyon 1, Lyon, France; 4 Virnext, Faculté de Médecine RTH Laennec, Université Claude Bernard Lyon 1, Université de Lyon, 69008 Lyon, France; 5 Vaxxel, 43 Boulevard du onze novembre 1918, 69100 Villeurbanne, France; 6 Université Paris-Saclay, INRAE, UVSQ, VIM, 78350 Jouy-en-Josas, France; 7 CIRI, Centre International de Recherche en Infectiologie, Team GIMAP, Université Claude Bernard Lyon 1, INSERM U1111, CNRS UMR5308, ENS Lyon, Université Jean Monnet Saint-Etienne, Saint-Etienne, France; 8 Nexomis, Faculté de Médecine RTH Laennec, Université Claude Bernard Lyon 1, Université de Lyon, 69008 Lyon, France

**Keywords:** Virology, Live attenuated vaccines, Viral infection

## Abstract

Live-Attenuated Vaccines (LAVs) stimulate robust mucosal and cellular responses and have the potential to protect against Respiratory Syncytial Virus (RSV) and Human Metapneumovirus (HMPV), the main etiologic agents of viral bronchiolitis and pneumonia in children. We inserted the RSV-F gene into an HMPV-based LAV (Metavac®) we previously validated for the protection of mice against HMPV challenge, and rescued a replicative recombinant virus (Metavac®-RSV), exposing both RSV- and HMPV-F proteins at the virion surface and expressing them in reconstructed human airway epithelium models. When administered to BALB/c mice by the intranasal route, bivalent Metavac®-RSV demonstrated its capacity to replicate with reduced lung inflammatory score and to protect against both RSV and lethal HMPV challenges in vaccinated mice while inducing strong IgG and broad RSV and HMPV neutralizing antibody responses. Altogether, our results showed the versatility of the Metavac® platform and suggested that Metavac®-RSV is a promising mucosal bivalent LAV candidate to prevent pneumovirus-induced diseases.

## Introduction

Human respiratory syncytial virus (RSV) and human metapneumovirus (HMPV) are two ubiquitous seasonal human pneumoviruses that cause frequent upper and lower respiratory tract infections (RTIs) throughout the globe^1^. Indeed, RSV infects >33 million people/year worldwide resulting in >3 million hospitalizations, with half occurring in infants under 6 months of age^1,2^. It is the main etiological agent of bronchiolitis and pneumonia in children younger than 1 year^1,3^, and causes up to 100,000 deaths in children under the age of 5^2^. The virus also constitutes an important health problem for adults over 60 and those with risk factors such as immunosuppression or pre-existing heart or lung diseases^4,5^. The other human pneumovirus, HMPV, is also a significant threat in the infant population, with >90% of children infected during their first 5 years of age^6^. It is responsible for 5–15% of hospitalizations following an acute lower RTI^7^ and particularly affects children between 1 and 3 years of age^8^. On the other hand, HMPV has been identified in 5–10% of adults or elderly people with an acute RTI and in 3–5% of adults having an exacerbation of chronic lung disease or community-acquired pneumonia^9,10^. In the US, the hospitalization rate for adults over 65 was reported to be 22 per 10,000 for HMPV, which is similar to RSV, with a rate of 25 per 10,000^9^.

The high prevalence of pneumovirus infections combined with the health and economic burden constitutes a major public health challenge in the face of the current limited therapeutic arsenal; as such, the WHO considers the development of vaccines against RSV as a priority^11^. Indeed, besides symptomatic measures (administration of oxygen or mechanical ventilation and bronchodilators/corticosteroids), a few specific prophylactic therapies are licensed such as Palivizumab (Synagis®) or Nirsevimab (Beyfortus®), human monoclonal antibodies against the RSV-F or RSV-preF protein respectively, that are used as passive immunization for high-risk infants to prevent severe forms of infection^12,13^.

Research in the RSV vaccine field was considerably slowed by safety concerns in the 1960s. A clinical trial that administered formalin-inactivated RSV vaccine led to enhanced pulmonary disease (EPD) in vaccinated infants upon subsequent RSV infection^14,15^. Moreover, natural infection with pneumoviruses leads to transient and non-protective immunity^16^, and reinfections occur throughout life^17^, with both RSV and HMPV having developed intrinsic strategies to counteract or skew the host’s immune responses^18–22^. More than 20 vaccine candidates against RSV are currently in clinical development^12,23,24^, including subunit vaccines, particle-based vaccines, chimeric viruses, mRNA, vector-based and live-attenuated vaccines (LAVs), and two subunit vaccines (GSK’s Arexvy® and Pfizer’s Abrysvo®) were recently approved by the FDA and/or EMA, for elderly or newborn immunization through maternal vaccination, respectively^25–27^.

While these strategies based on stabilized pre-fusion F protein are reported to reduce the risk of developing severe disease, one of the limitations of such an intramuscularly (IM)-delivered vaccine resides in its poor capacity to induce a protective and persistent mucosal immune response able to block the transmission of respiratory viruses. With this objective, LAVs against respiratory viruses administered by intranasal (IN) route are considered a strategy of choice for the pediatric population^28^, as they mimic natural viral infection while eliciting robust mucosal and cellular responses without requiring adjuvant^28,29^. Moreover, IN-delivered LAVs offer several advantages over IM-administered vaccines, being easy to use, non-invasive, and more adapted for mass vaccination. About a dozen of LAV candidates against RSV are currently in clinical development and four of them have progressed to phase 2 evaluation^30,31^. Attenuation was achieved by deletion or modification of the NS2, SH, G, or M2-2 genes, and/or inserting temperature sensitivity mutations in the L gene^30,32–37^. Several evaluations of LAV candidates confirmed the safety of this vaccine strategy, without associated enhanced respiratory disease; however, variable immunogenicity (neutralizing antibody and mucosal IgA responses) and duration of protection have been reported. Furthermore, the pre-existing serology status of adults and children can affect the clinical outcomes^30,34–36,38–40^. No LAVs against HMPV are currently in clinical development, but some attenuated pneumoviruses with G and/or SH gene deletions have shown the potential to progress toward clinical stages^35,41–43^.

We have previously developed an LAV platform (Metavac®) based on a recombinant HMPV A1/C-85473 strain expressing an endogenous hyperfusogenic F protein and attenuated by deletion of its SH gene (ΔSH-rC-85473-GFP)^44,45^. We provided evidence that such a deletion in the C-85473 backbone prevents the virus-induced activation of the NLRP3-inflammasome, subsequently reduces lung inflammation, and attenuates pathogenicity in HMPV-infected mice^44,46^. We also described that vaccination of mice with Metavac® confers protection against lethal homologous HMPV A challenge, stimulates the induction of neutralizing antibody responses against homologous and heterologous HMPV strains, and reduces lung inflammatory response without detectable markers of enhanced disease^44^.

In this context, we re-engineered the Metavac® LAV candidate by reverse genetics and rescued a replicative and stable bivalent attenuated virus (Metavac®-RSV) expressing a native fusion protein of RSV A2 (RSV-F) in addition to its own HMPV-F. Using transmission electron microscopy (TEM), immunostaining, and flow cytometry assays, we confirmed the efficient expression of both RSV and HMPV-F proteins at the virus particle surface, in infected monolayers of LLC-MK2 cells, and in the human airway epithelium (HAE) model. This prompted us to administrate Metavac®-RSV to BALB/c mice by IN route to evaluate its capacity to induce neutralizing antibody responses and protection against RSV A and lethal HMPV A challenges. Our results suggest that Metavac® is a versatile LAV platform and that Metavac®-RSV is a promising mucosal bivalent LAV candidate to prevent bronchiolitis and severe pneumonia induced by pneumoviruses.

## Results

### Rescue and in vitro characterization of the bivalent Metavac®-RSV virus

**Fig. 1:**
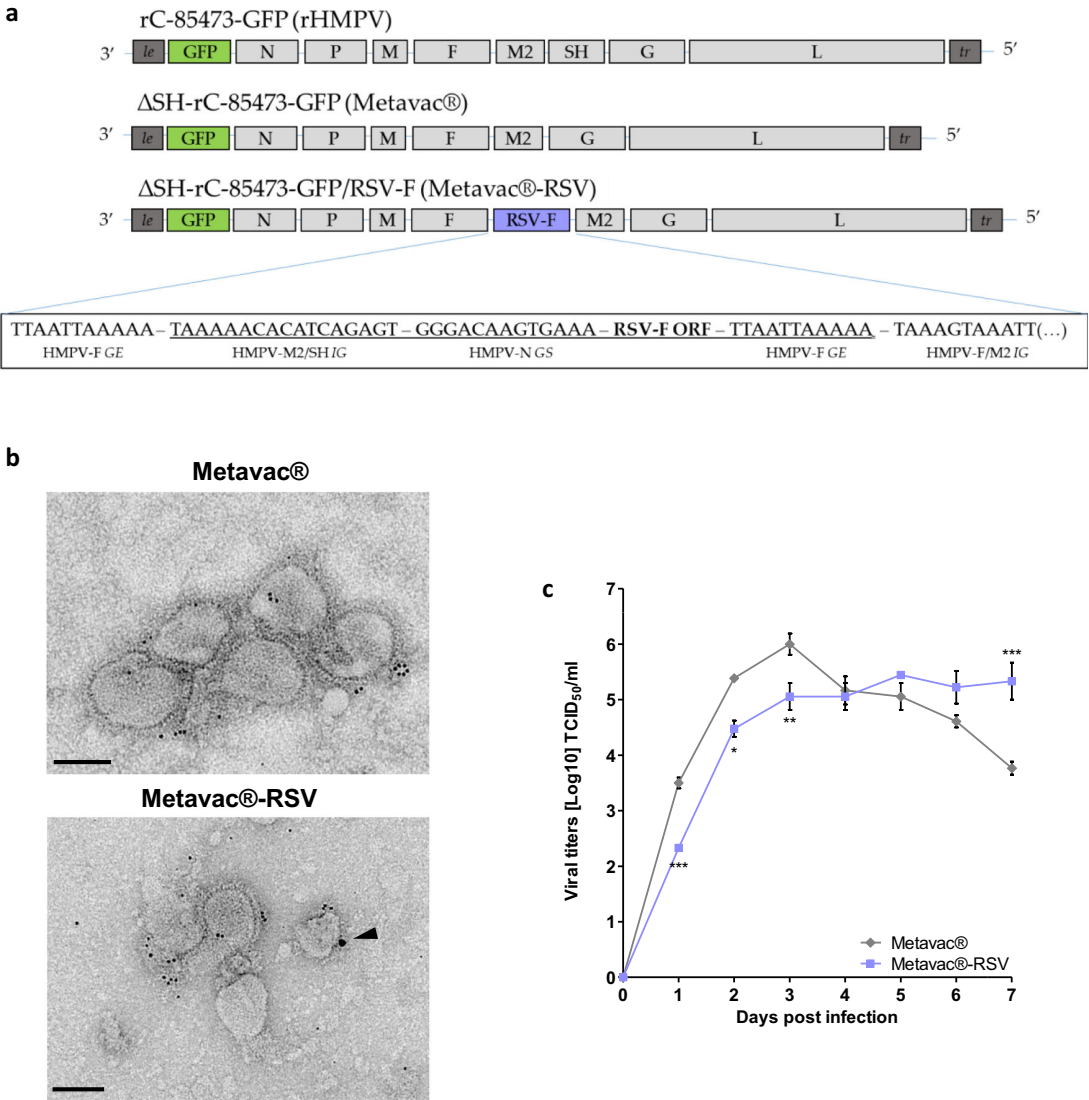
Rescue and characterization of recombinant Metavac®-RSV virus. **a** Schematic genomic organization of the recombinant HMPV strain (rC-85473-GFP, rHMPV), monovalent Metavac® (ΔSH-rC-85473-GFP), and bivalent Metavac®-RSV (ΔSH-rC-85473-GFP/RSV-F) viruses is represented and the insertion site of the RSV-F ORF between HMPV-F and M2 genes in Metavac®-RSV genome is detailed. GS - Gene Start, GE - Gene End, IG - intergenic sequence. Sequences added to the ΔSH-rC-85473-GFP genome are underlined. Genomic sequence is presented from 3′ to 5′. **b** After the viral rescue, in vitro expression of the RSV-F protein at the surface of Metavac®-RSV viral particles was visualized by transmission electron microscopy after immunogold labeling with anti-HMPV serum (5 nm bead) and the Palivizumab (15 nm bead, black arrowhead) Scale bar = 100 nm. **c** Viral replication kinetics of the Metavac®-RSV virus were measured in LLC-MK2 cells and compared to the monovalent Metavac® counterpart. Over a 7-day period, culture supernatants were harvested and titrated in TCID_50_/ml. Results represent the mean of 3 experimental replicates for each time-point ± SD. *p < 0.05, **p < 0.01, ***p < 0.001 when comparing Metavac®-RSV to Metavac® virus using repeated measures two-way ANOVA.

After reporting the advantageous properties of the Metavac® (ΔSH-rC-85473-GFP) recombinant virus and its potential as an LAV candidate against HMPV A and B strains^44^, we sought to enlarge the protective scope of this vaccine platform by adding the expression of the exogenous RSV-F antigen. To generate such a recombinant virus by reverse genetics, we inserted the coding sequence of the RSV A2 fusion protein into the intergenic region between F and M2 genes in the pSP72 plasmid containing the complete antigenome sequence of Metavac®, as detailed in Fig. 1a. Insertion in the F/M2 junction resulted in the rescue of a replicative virus (Metavac®-RSV) that was successfully amplified by passages in LLC-MK2 cells, following standard recombinant HMPV rescue protocols^47^.

By using TEM, we then visualized pleiomorphic virus particles covered with glycoproteins in viral suspensions, in the shape and size concordant with that known for the HMPV virus and similar to the Metavac® virus (Fig. 1b). Using dual immunogold labeling, we also detected the RSV-F protein on the surface of Metavac®-RSV virions, in addition to the endogenous HMPV proteins (Fig. 1b), demonstrating that the insertion of RSV-F ORF resulted in efficient gene expression, protein production and, ultimately, to the incorporation of the RSV-F protein into the membrane of the viral particles. We then characterized the daily growth kinetics of the Metavac®-RSV virus in LLC-MK2 cells over a 7-day period (Fig. 1c). In comparison to the Metavac® virus, which peaked at 6 ± 0.33 log10 TCID_50_/ml after 3 dpi, the Metavac®-RSV virus had a slower viral replication, reaching a peak of 5.44 ± 0.09 log10 TCID_50_/ml after 5 dpi (Fig. 1c).

**Fig. 2:**
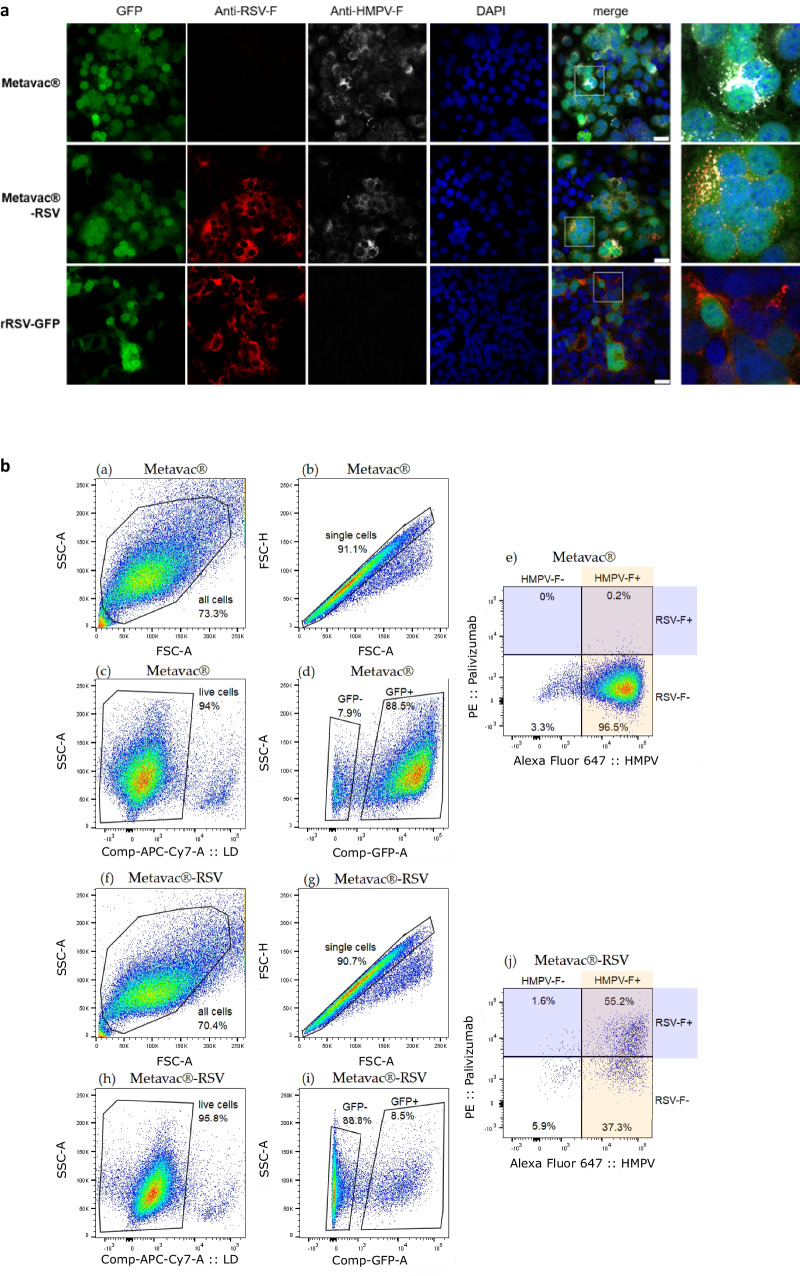
Co-immunostaining of HMPV and RSV-F glycoproteins in infected LLC-MK2 cells. **a** LLC-MK2 cells were infected with GFP-expressing Metavac®, Metavac®-RSV or RSV (rRSV-GFP) viruses, fixed and stained at 3 dpi with Palivizumab (red), HMPV24 mAb (white) and DAPI (blue). Merged fluorescent signals are represented (yellow). Images of representative cytopathic effects (CPEs) were taken using Zeiss880 confocal microscope (×40 magnification) and processed with ImageJ software. Scale bar = 25 µm. A numeric focus was made on CPEs (square) and presented in the right panel. **b** LLC-MK2 cells were infected with MOI 0.5 of either Metavac® (**a**–**e**) or Metavac®-RSV (**f**, **j**), and antigens expression on the surface of the infected cells was measured by flow cytometry 48 h post-infection. HMPV-F protein was detected with HMPV24 mAb conjugated with Alexa Fluor™ 647 and RSV-F protein was detected with Palivizumab conjugated with R-Phycoerythrin. Cells were sorted and analyzed by LSR II Flow Cytometer (BD biosciences®). Approximately 30,000 single live cells were counted per each sample performed in triplicate. The figure shows representative gating of sub-populations on one of the three samples. **a**, **f** all cells in the sample; **b**, **g** single cells **c**, **h** single live cells **d**, **i** single live cells positive or negative for GFP expression **e**, **j** the percentage of GFP-expressing infected single live cells with HMPV-F expression revealed by HMPV24 mAb and RSV-F expression revealed by Palivizumab.

To further investigate the expression of both endogenous HMPV-F and exogenous RSV-F fusion proteins on the cell surface of infected LLC-MK2 cells, we performed co-immunostaining to visualize and measure those antigens by confocal fluorescent microscopy and flow cytometry. Expression of GFP reporter protein showed that the Metavac®-RSV virus harbored a hyperfusogenic phenotype (Fig. 2a), according to previous studies with the viral C-85473 background^47,48^. Co-immunostaining with anti-RSV-F (Palivizumab) and anti-HMPV-F (HMPV24) mAbs confirmed the co-expression of the RSV-F protein together with that of HMPV-F by infected cells. When focusing on multinucleated cells at 3 dpi, the merged fluorescent signal suggested that RSV-F and HMPV-F proteins were colocalized (Fig. 2a). To validate this observation, flow cytometry using Palivizumab and HMPV24 mAbs was performed to measure the proportion of infected LLC-MK2 cells expressing both of the antigens at the cell surface 48 h post-infection (hpi, Fig. 2b). Despite low quantity of infected cells (GFP+) with Metavac®-RSV compared to Metavac®, we confirmed that 55.2% of the cells infected with Metavac®-RSV co-expressed both HMPV-F and RSV-F proteins at their surface, whereas 37.3% and 1.6% of infected cells only expressed HMPV-F or RSV-F proteins, respectively (Fig. 2b). As a comparison, 96.5% of cells infected with Metavac® only expressed HMPV-F protein at their surface. To verify the stability of F-RSV gene or protein expression in the context of the Metavac®-RSV bivalent virus, we performed 10-serial cell passages of Metavac® and Metavac®-RSV on LLC-MK2 cells, and we analyzed viral genome by deep RNA sequencing and protein expression by immunostaining. We identified a limited number of non-synonymous mutations (13 for Metavac® and 14 for Metavac®-RSV). In particular, we identified a mutation leading to a loss of stop codon can occur in the F-RSV gene, which is compensated by the emergence of a new stop codon, resulting in an extension of only 7 amino acids in the intracellular domain of the glycoprotein (Supplementary Fig. 1). We then performed immunostaining on P10 Metavac-RSV infected cells with HMPV-F mAb and RSV-F Palivizumab and confirmed that F-RSV is still expressed and recognized by Palivizumab after 10 passages of Metavac®-RSV (Supplementary Fig. 2), underscoring the robustness of the bivalent vaccine design with regard to minor genomic alterations that are common in RNA viruses.

Altogether, these results show that the insertion of the RSV A2-F coding sequence into the Metavac® genome is viable and stable over multiple virus replication cycles, it results in the production of a mild-attenuated bivalent Metavac®-RSV virus and leads to the efficient expression of both F-HMPV and F-RSV proteins.

### Bivalent Metavac®-RSV virus infects and expresses the RSV-F protein at the apical pole of the HAE model

**Fig. 3:**
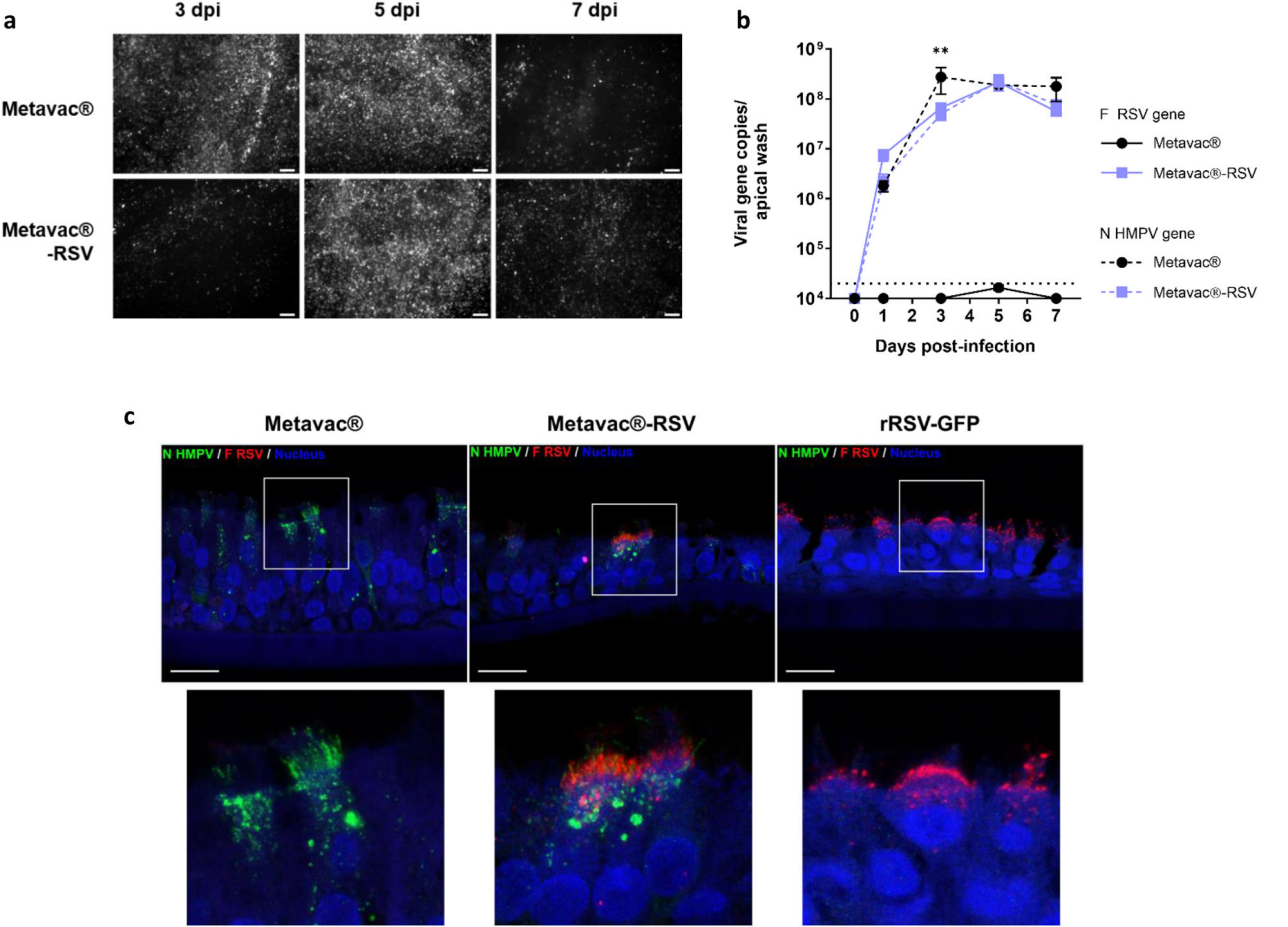
Viral replication and RSV-F expression in human airway epithelium (HAE) model. Reconstituted HAEs were infected with Metavac® or Metavac®-RSV viruses at an MOI of 0.1 and monitored for 7 days. **a** Viral spread in HAE was monitored at 3, 5, and 7 dpi by GFP fluorescence observation (10× magnification). Scale bar = 100 µm. **b** Viral quantification from epithelium apical washes collected after 1, 3, 5, and 7 dpi was performed by RT-qPCR targeting the HMPV-N gene or the RSV-F gene. Data are shown as means ± SD and represent experimental triplicates. The dotted line represents the RT-qPCR quantification threshold. **p < 0.01 when comparing Metavac®-RSV N-HMPV gene expression to Metavac® virus using repeated measures two-way ANOVA. **c** Co-immunostaining of HMPV-N and RSV-F proteins was performed at 3 dpi. HAE infected by Metavac®, Metavac®-RSV, or rRSV-GFP viruses were fixed and cross-sections were stained with a mixture of mAbs specific to the HMPV-N protein (mAb hMPV123, green), RSV-F protein (Palivizumab, red) and with DAPI (blue) specific to the nucleus. Acquisition of images of representative infected areas was performed with confocal inverted microscope (Zeiss Confocal LSM 880) and processed with ImageJ software. Scale bar = 20 µm. A focus on the apical surface of ciliated infected cells was made (square) and is presented in the right panel.

We further assessed the properties of the bivalent Metavac®-RSV virus to infect and replicate in reconstituted HAE. Indeed, we previously showed that Metavac® behaves very similarly to its non-∆SH rHMPV counterpart, mimicking the in vivo host respiratory epithelium response to such infection^44^. In line with these results, we observed that the Metavac®-RSV virus was still infectious and spread within the HAE model, as illustrated by the propagation of the GFP signal during 7-day replication kinetics (Fig. 3a), which appeared somewhat slower than the propagation of the Metavac® virus (Fig. 3a). To confirm the delay in virus propagation observed by fluorescent microscopy, we quantified the viral genome present at the apical surface of such HAE and measured a peak number of HMPV-N gene copies at 3 dpi for Metavac® (2.72 × 10^8^) and 5 dpi for Metavac®-RSV (2.26 × 10^8^) (Fig. 3b). Following our previous results, we also demonstrated that the Metavac®-RSV virus expressed its exogenous RSV-F gene in an amplification pattern concomitant to the HMPV-N gene expression, reaching a peak of 2.14 × 10^8^ copies of RSV-F gene per apical wash at 5 dpi (Fig. 3b).

We then asked whether and where the expression of the exogenous RSV-F protein was localized within the infected HAE. We thus performed immunofluorescence staining of both RSV-F and HMPV-N proteins at 3 dpi (Fig. 3c). By our knowledge of the pneumovirus replication cycle^44,45,49,50^, we observed in Metavac®-infected HAE that the HMPV-N protein was localized in large areas in the cytoplasm of ciliated cells, presumably inclusion bodies corresponding to viral replication, as well as into the cilia, where new virions bud from the cell membrane (Fig. 3c). When infected with Metavac®-RSV, ciliated cells positive for HMPV-N expression also expressed the RSV-F protein observed by Palivizumab staining and that mainly localized into the apical ciliated surface and into smaller cytoplasmic speckles, similar to what was observed when HAEs were infected with rRSV-GFP virus (Fig. 3c).

Taken together, these results indicate that Metavac®-RSV has a mild-attenuated phenotype in HAE (Fig. 3), as well as in LLC-MK2 cells (Fig. 1), in comparison with its monovalent Metavac® counterpart. However, we confirmed that Metavac®-RSV is characterized by efficient infection, spreading, and RSV-F protein expression in the HAE model, encouraging further investigation of its potential as a bivalent LAV candidate in vivo.

### Infectivity and attenuation of Metavac®-RSV vaccine candidate in BALB/c mice

**Fig. 4:**
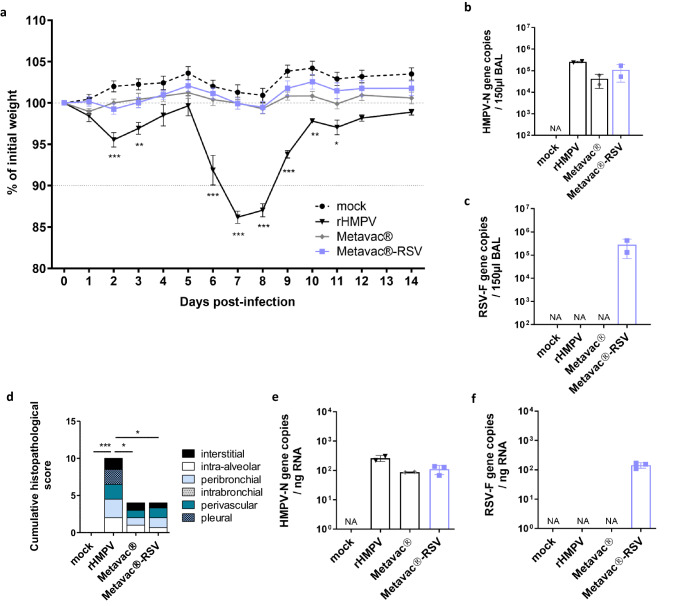
Viral growth and attenuation of the Metavac®-RSV vaccine candidate in BALB/c mice. BALB/c mice were infected by the IN route with 5 × 10^5^ TCID_50_ of rHMPV virus, Metavac®, or Metavac®-RSV vaccine candidates. **a** Weight loss was monitored for 14 dpi (*n* = 16). Data are shown as means ± SEM. *p < 0.05, **p < 0.01, ***, *p* < 0.001 when compared to mock-infected mice using Repeated Measures Two-way ANOVA. **b**, **c** At 2 dpi, mice were euthanized, and BALs were harvested to measure HMPV-N gene (**b**) or RSV-F gene (**c**) copies by RT-qPCR (*n* = 2). **d** At 5 dpi, mean cumulative histopathological scores (peribronchial, intrabronchial, perivascular, interstitial, pleural, and intra-alveolar inflammation scores) of the lungs from infected mice were evaluated (*n* = 3). *p < 0.05, ***p < 0.001 when comparing mean global histopathological score to mock-infected mice using One-way ANOVA. **e**, **f** At 5 dpi, HMPV-N (**e**) or RSV-F (**f**) gene copies were measured by RT-qPCR from total RNA extracted from fixed lung tissues (*n* = 2–3). Data of viral gene quantification are shown as means ± SD.

To ascertain the level of the Metavac®-RSV attenuation in vivo, we infected BALB/c mice by the IN route with 5 × 10^5^ TCID_50_ of either rHMPV, Metavac®, or Metavac®-RSV viruses. As previously reported^44^, this represents a non-lethal dose shown to induce significant weight loss after rHMPV infection but not with Metavac® infection. We observed neither weight loss nor clinical signs when mice were infected with the Metavac®-RSV vaccine candidate, similar to the mock (non-infected) group control, while the rHMPV virus did cause weight loss, which confirms an attenuated phenotype in BALB/c mice (Fig. 4a).

To measure viral replication in the respiratory tract of infected mice, we collected bronchoalveolar lavages (BALs) 2 days after IN instillation and quantified viral genome copies by RT-qPCR. We found no significant difference in HMPV-N gene copy number in animals infected with rHMPV, Metavac®, or Metavac®-RSV viruses, i.e., 2.5 × 10^5^, 4.2 × 10^4^ or 1.1 × 10^5^, respectively (Fig. 4b), and we found comparable levels of N-HMPV and F-RSV gene expression only in animals infected with Metavac®-RSV (Fig. 4c).

At 5 dpi, mice were euthanized to investigate inflammatory profiles by histopathological scoring of lung compartments. In agreement with the weight curves, mean cumulative histopathological scores were significantly lower after infection with either Metavac® or Metavac®-RSV, compared to the rHMPV group (the scores of 4 and 4 versus 10, respectively), owing primarily to the absence of pleura inflammation and reduction in peribronchial, perivascular and interstitial inflammation (Fig. 4d and Supplementary Fig. S3). We then extracted total RNA from fixed paraffin-embedded lung tissues to estimate pulmonary viral load by RT-qPCR at 5 dpi. We quantified a mean number of 2.61 × 10^2^, 8.7 × 10^1,^ or 1.1 × 10^2^ of HMPV-N gene copies in the lungs of mice infected with either rHMPV, Metavac®, or Metavac®-RSV, respectively (Fig. 4e). In agreement with the viral detection in BALs at 2 dpi, we also detected a mean number of 1.4 × 10^2^ RSV-F gene copies in mice infected with the Metavac®-RSV virus (Fig. 4f).

Altogether, we validated that the Metavac®-RSV vaccine candidate replicates in the pulmonary airways of infected mice after intranasal instillation, and induces attenuated pathology, characterized by the absence of weight loss and reduced inflammatory profile, similar to the monovalent Metavac® LAV candidate.

### Bivalent Metavac®-RSV vaccine candidate protects mice against lethal HMPV challenge

**Fig. 5:**
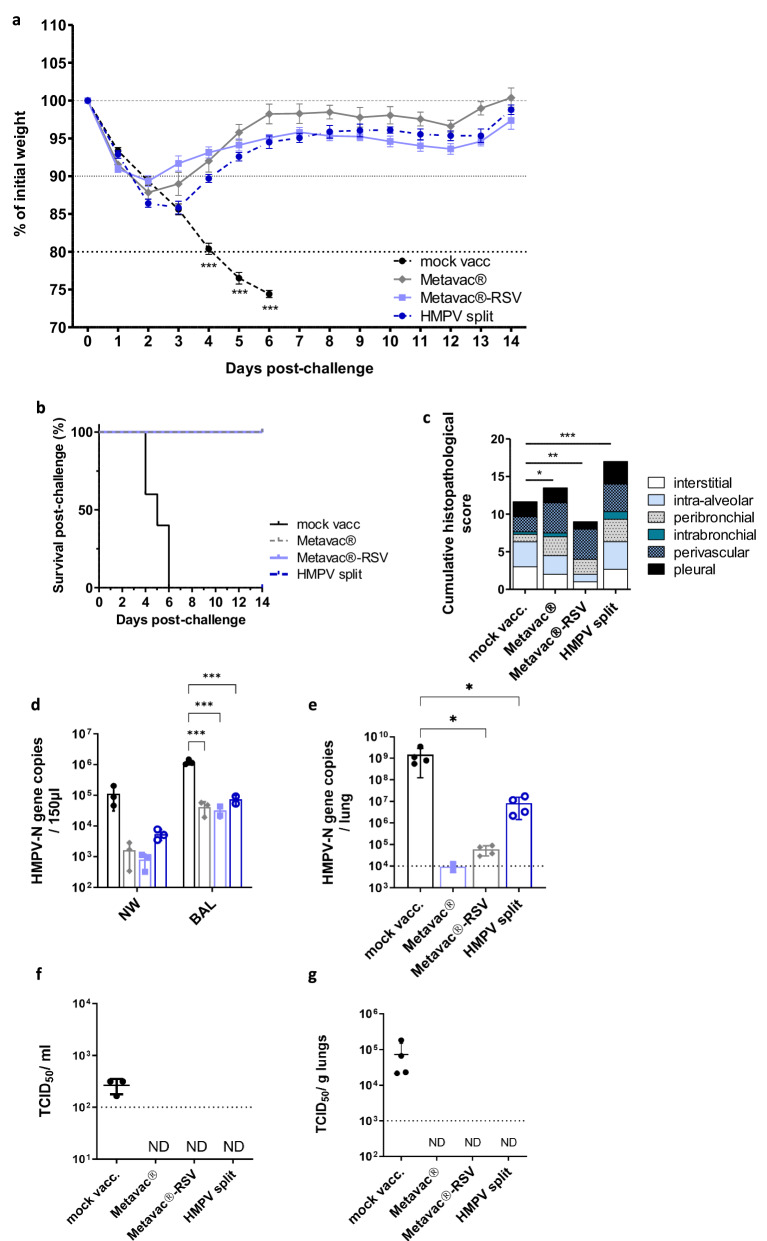
Efficacy of Metavac®-RSV vaccine candidate against lethal challenge with HMPV. BALB/c mice were immunized twice with a 21-day interval by the IN route with 5×10^5^ TCID_50_ of Metavac® or Metavac®-RSV LAV candidates or by the IM route with HMPV split preparation adjuvanted with AddaVax™. Three weeks after the last immunization, animals (*n* = 12/group) were inoculated with a lethal dose of the rHMPV virus. **a** Weight loss and **b** mortality rates were monitored for 14 days post-challenge (dpc, *n* = 8/group). Data are shown as means ± SEM. ***p < 0.001 when comparing to Metavac® vaccinated mice using Two-way ANOVA. **c** At 5 dpc, cumulative pulmonary histopathological scores (peribronchial, intrabronchial, perivascular, interstitial, pleural, and intra-alveolar inflammation scores) were also evaluated (*n* = 3/group). **d** At 2 dpc, mice were euthanized and nasal washes (NW) and bronchoalveolar lavages (BALs) were harvested to measure HMPV-N gene copies by RT-qPCR (*n* = 2/group). **e** At 5 dpc, RT-qPCR was performed on total RNA recovered from mouse lung homogenates (*n* = 4/group) to quantify HMPV-N gene copies. **f**, **g** Infectious TCID_50_ titers were measured from BAL samples collected at 2 dpc (**f**) or lung homogenates collected at 5 dpc (**g**). Data are shown as means ± SD. *p < 0.05, **p < 0.01, ***p < 0.001 when comparing mean global histopathological score to mock-vaccinated mice using one-way ANOVA.

We then sought to characterize the immunogenicity and protection conferred by the Metavac®-RSV bivalent LAV candidate against HMPV viral challenge in the mouse model. BALB/c mice were immunized twice with a 21-day interval by the IN route with 5 × 10^5^ TCID_50_ of Metavac® or Metavac®-RSV vaccine candidate, or by the IM route with inactivated HMPV split adjuvanted with AddaVax™ before viral challenge with a lethal dose of rHMPV virus three weeks after the last immunization, as previously described^44^. The choice of a two-immunization scheme is well adapted to the use of naïve animals, as it has the advantage of enabling the evaluation of the immunogenicity after only one immunization (day 20) but is also informative on a putative booster effect of a second vaccine dose (day 41). Upon viral challenge with 2 × 10^6^ TCID_50_ of rHMPV virus, mock-immunized mice showed a 100% HMPV-associated mortality at 6 days post-challenge (dpc), as expected (Fig. 5a, b). On the other hand, all three vaccinated groups showed complete protection from rHMPV-associated mortality (Fig. 5b) and weight loss, with a maximum loss of 10.7%, 12.2%, and 14.2% at 2 or 3 dpc when vaccinated with Metavac®-RSV, Metavac® or HMPV split, respectively (Fig. 5a).

After the viral challenge, non-immunized mice developed interstitial pneumonia of moderate intensity with a minimal-to-mild peribronchial and perivascular inflammation with pulmonary edema, corresponding to a mean total histopathological score of 11.66 (Fig. 5c and Supplementary Fig. S3). In comparison, the group of animals vaccinated with Metavac®-RSV virus had a reduced total inflammatory score of 9 with a significantly milder inflammation in the interstitial compartment. Animals vaccinated with Metavac® showed a mean histopathological score of 13.5 owing to peri-bronchial and perivascular inflammation, while interstitial pathology was slightly reduced in this group when compared to the non-vaccinated mice. In contrast, animals vaccinated with the HMPV split showed the highest total histopathological score (mean score of 17) with moderate-to-marked changes in all the compartments, as well as eosinophil, lymphocyte, and macrophage infiltration around bronchi, in alveoli and around the blood vessels (Fig. 5c and Supplementary Fig. S3). Overall, after the infectious challenge, Metavac® and Metavac®-RSV-vaccinated animals showed signs of pulmonary inflammation, although not associated with interstitial pneumonia or exaggerated reaction, as induced by the IM administration of HMPV split.

To evaluate the vaccination efficacy, we then measured viral genome and infectious titers from respiratory tract lavages and lung tissues. In line with weight curves, we detected significantly reduced levels of viral gene copies from nasal washes (NW) and BALs in any of the three different immunized groups at 2 dpc, in contrast to 10- to 100-fold more viral gene copies in the mock-vaccinated group (Fig. 5d). Moreover, N-HMPV copy numbers in lungs were reduced by 4 or 5 log10 in animals vaccinated with Metavac®-RSV or Metavac® LAV candidates at 5 dpc, respectively, compared to mock-vaccinated animals (Fig. 5e). In contrast, animals vaccinated with HMPV split showed a reduction in the viral genome of only 100-fold compared to mock, suggesting that Metavac®-RSV and Metavac® LAV candidates administered by the IN route are more efficient in inhibiting viral replication in the lower respiratory tract (Fig. 5e). We also confirmed that no RSV-F gene copies were detected in these tissues, showing that replicative Metavac®-RSV used for the vaccination was eliminated from the lungs at the time of the viral challenge (data not shown). Following these results, we were able to titrate infectious HMPV virus only from BALs (Fig. 5f) and lung (Fig. 5g) samples collected from non-immunized mice.

**Fig. 6:**
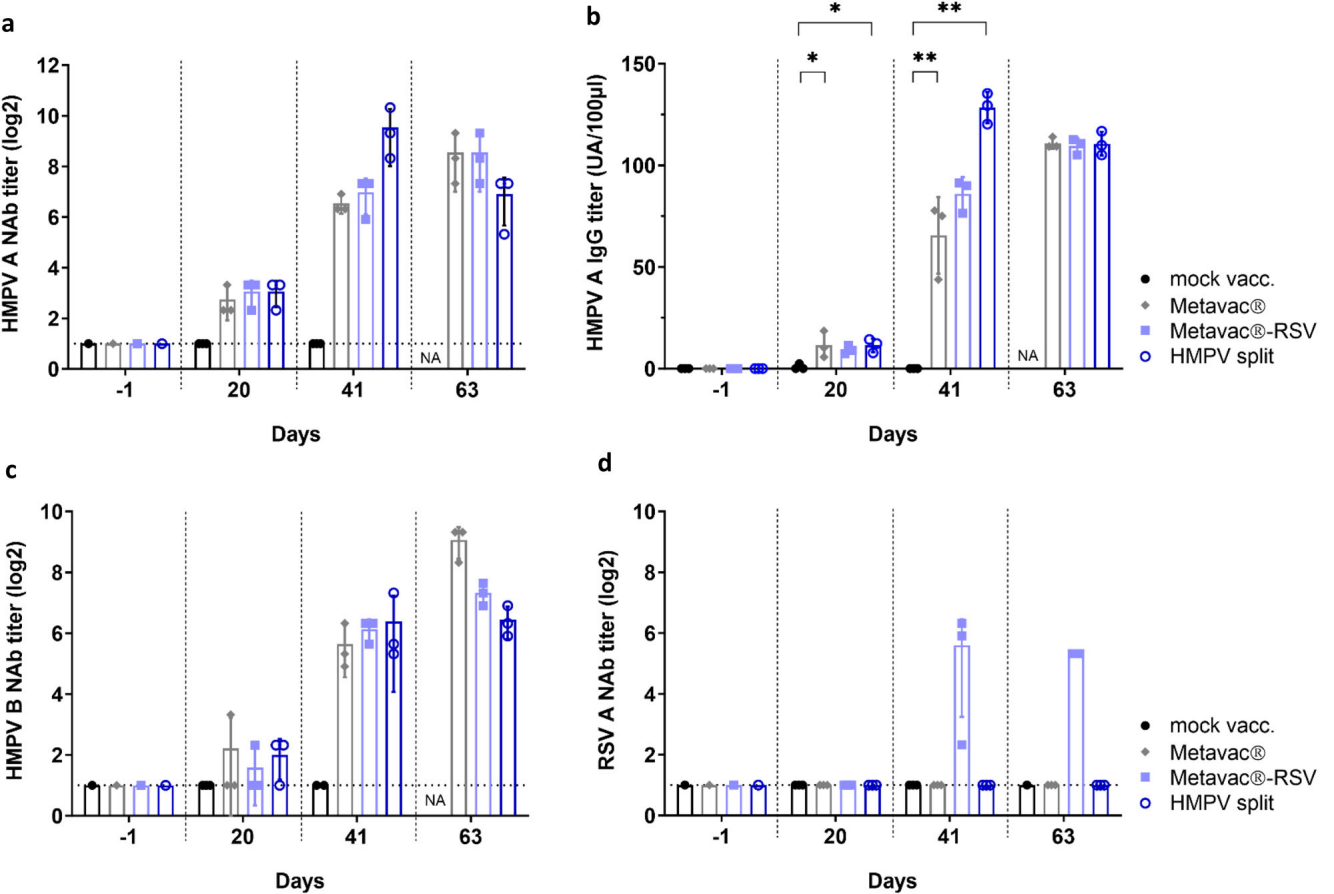
Immunogenicity of Metavac®-RSV vaccine candidate before and after lethal challenge with HMPV. BALB/c mice were immunized twice with a 21-day interval by the IN route with 5 × 10^5^ TCID_50_ of Metavac® or Metavac® -RSV vaccine candidates or by the IM route with the adjuvanted HMPV split preparation. Three weeks after the last immunization, animals (*n* = 12/group) were inoculated with a lethal dose of rHMPV. Immunogenicity of vaccine candidates was measured at −1, 20, 41, or 63 days after the first immunization by microneutralization (**a**, **c**, **d**) or ELISA (**b**) assays from pools of sera (*n* = 3 pools/group). Neutralization titers were defined by an endpoint dilution assay based on fluorescent detection of (**a**) HMPV A, (**c**) HMPV B, or (**d**) RSV A and represented as mean log2 reciprocal neutralizing antibody (NAb) titers. **b** IgG titer specific to HMPV A virus was represented as an arbitrary unit based on endpoint absorbance. Naive status of mice was confirmed by processing the samples harvested one day before vaccination. Data are shown as means ± SD. *p < 0.05, **p < 0.01 when comparing each vaccinated group to the mock-vaccinated condition using Two-way ANOVA.

We then investigated the levels of circulating neutralizing antibodies (NAb) and HMPV-specific IgG for the different vaccinated groups compared to mock-vaccinated animals (Fig. 6). Immunization with Metavac®-RSV was associated with a progressive increase in NAb levels reaching the highest titers at 21 dpc (63 days apart from the first immunization), similar to the Metavac®-vaccinated group (Fig. 6a). As expected, we detected significant levels of anti-HMPV-specific IgG in vaccinated groups compared to the non-immunized animals, following the kinetics of NAbs induction (Fig. 6b). Interestingly, Metavac®-RSV-vaccinated animals also showed the production of NAbs against a heterologous HMPV B strain (Fig. 6c), similarly to Metavac®-vaccinated mice, and NAbs against a RSV A virus (Fig. 6d), demonstrating its ability to induce a broad immune response in vaccinated animals which persisted after HMPV challenge.

### Bivalent Metavac®-RSV vaccine candidate protects mice against the RSV challenge

**Fig. 7:**
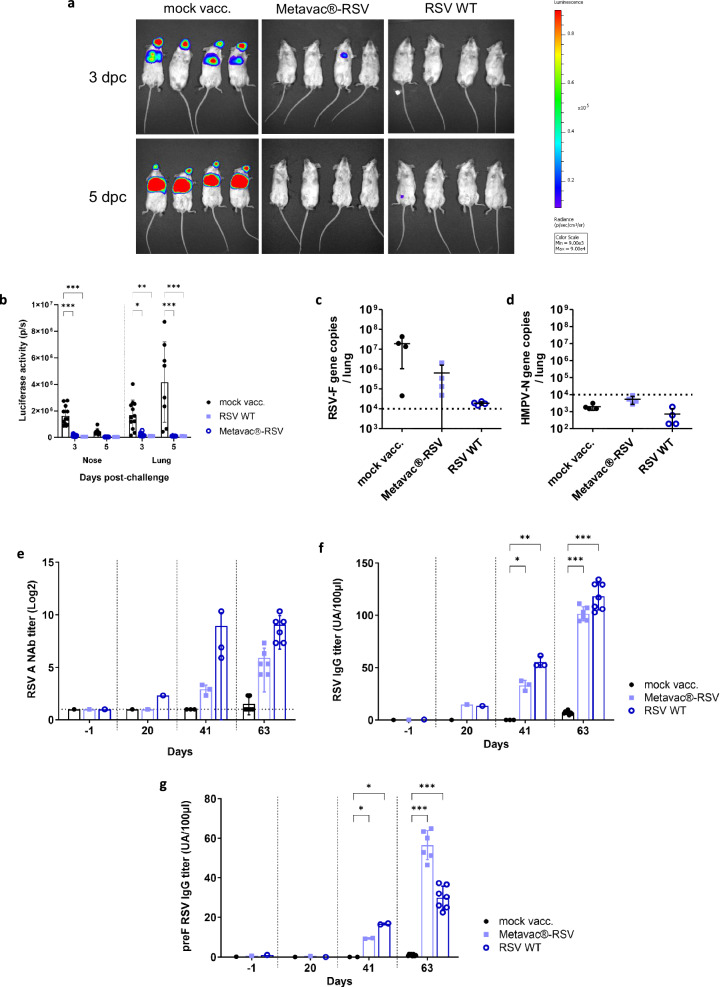
Efficacy and immunogenicity of Metavac®-RSV vaccine candidate following RSV challenge. BALB/c mice were immunized twice with a 21-day interval by the IN route with 5 × 10^5^ TCID_50_ of Metavac**®**-RSV vaccine candidate or rRSV-mCh (RSV WT) virus. Three weeks after the last immunization, animals (*n* = 12/group) were inoculated with 1 × 10^5^ PFU of rRSV-Luc virus. **a**, **b** Bioluminescence was measured at 3 and 5 dpc by IN injection of 50 µl of D-Luciferin (200 mM). **a** Ventral views of 4 representative mice were taken using the IVIS system. The scale on the right indicates the average radiance (a sum of the photons per second from each pixel inside the region of interest, ps-1 cm-2 sr-1). **b** Luciferase activities were quantified using ‘Living Image’ software and were represented as mean ± SEM photons per second (p/s) (*n* = 8/group). **c**, **d** RT-qPCR was performed on total RNA recovered from mouse lung homogenates (n = 4/group) harvested at 4 dpc to quantify RSV-F (**c**) or residual HMPV-N gene copies (**d**). **e**–**g** Immunogenicity of the Metavac**®**-RSV LAV candidate was measured by RSV A microneutralization assay, anti-total RSV or anti-preF RSV IgG ELISA assays from pools of sera (before each IN instillation at −1, 20, and 41 dpi) or individual sera (at the endpoint at 63 dpi, *n* = 6–8). **e** Neutralization of RSV A strain was represented as mean log2 reciprocal NAb titer. **f**, **g** IgG titer specific to RSV virus (**f**) or recombinant preF RSV protein (**g**) was represented as an arbitrary unit based on endpoint absorbance. Data are shown as means ± SD. *p < 0.05, **p < 0.01, ***p < 0.001 when comparing Metavac**®**-RSV or RSV WT vaccinated group to the mock-vaccinated condition using Two-way ANOVA.

Finally, we analogously sought to characterize the immunogenicity and protection conferred by the Metavac®-RSV bivalent LAV candidate against RSV viral challenge in mice. As previously, we immunized BALB/c mice twice with a 21-day interval by the IN route with 5 × 10^5^ TCID_50_ of Metavac-RSV and then challenged mice with rRSV-Luc virus in order to compare the efficacy of the Metavac®-RSV LAV candidate to groups of mock-vaccinated mice or those vaccinated with RSV WT virus, using rRSV-mCh as a surrogate. Following the challenge with 1 × 10^5^ PFU of rRSV-Luc, the viral replication in the upper and lower respiratory tract of infected animals could be visualized by an in vivo imagery system revealing luciferase expression. The images and measures taken at 3 or 5 dpc showed a progressive intensification in the in vivo bioluminescence activity, representing increased viral replication in the lung tissue, and a constant viral replication in the nasal compartment of mock-vaccinated mice (Fig. 7a, b). Indeed, the bioluminescence measured 5 dpc in mice vaccinated with Metavac®-RSV LAV candidate or RSV WT was significantly reduced in the upper and lower respiratory tracts (Fig. 7a), with a cumulative luciferase activity of 1.25 × 10^5^ ± 15 700, and 1.10 × 10^5^ ± 8 400 photons per second, respectively, in comparison to 4.6 × 10^6^ ± 2.9 × 10^6^ photons per second for mice in the mock-vaccinated group (Fig. 7b). On 4 dpc, mice were euthanized, and viral lung titers were measured by RT-qPCR. In these samples, we observed mean lung viral titer reductions of 10-fold and 1000-fold in animals vaccinated with Metavac®-RSV or RSV WT viruses, respectively, compared to mock-vaccinated animals (Fig. 7c). As previously, we validated that no residual HMPV-N gene copies from IN vaccinations were detected after the RSV challenge in Metavac®-RSV-vaccinated animals (Fig. 7d).

Lastly, we measured the level of circulating NAb and IgG against RSV in samples from vaccinated mice (Fig. 7e–g). In comparison to the mock-vaccinated animals, the animals vaccinated with Metavac®-RSV LAV candidate developed high NAb titers after the viral challenge, similar to those observed in the group vaccinated with RSV WT (Fig. 7e). As previously observed with anti-HMPV IgG induction, we measured significant levels of anti-RSV-specific IgG with a maximal titer measured 21 dpc, following NAb kinetics along the timeline (Fig. 7f). It is known that RSV NAbs are mostly directed against epitopes presented in the pre-fusion form of the F protein (preF)^51^, we also measured by ELISA the specific preF IgG titer and we confirmed that the Metavac®-RSV vaccination induced a strong IgG response with preF affinity (Fig. 7g). Importantly, we also measured NAb titers directed against contemporary RSV A and B strains and we confirmed neutralizing responses against both RSV subtypes in animals vaccinated with Metavac®-RSV and RSV WT virus after challenge (Supplementary Fig. S4).

Hereby, we demonstrated that the Metavac®-RSV LAV candidate administered by the IN route efficiently protects vaccinated mice against both HMPV and RSV challenges, restraining viral replication in the pulmonary tract of the animals and inducing a broad immune response, characterized by high titers of circulating NAb and specific IgG against both HMPV and RSV.

## Discussion

Despite over 60 years of research in the field of anti-RSV vaccine, a limited number of vaccine candidates have moved to clinical phases in humans^30^. Subunit, mRNA, and vectored vaccine candidates are currently the most advanced strategies for maternal or elderly vaccination. In May 2023, the GSK’s vaccine (Arexvy®), a recombinant stabilized pre-fusion F protein combined with the ASO1 adjuvant, was the first vaccine approved by the FDA to prevent severe RSV disease in the elderly population^25^. In contrast, the development of pediatric vaccines is still ongoing. Several LAV candidates have progressed to clinical trials in infants. LAVs can induce local mucosal immune responses through their administration by the IN route, in addition to strong T-cell responses. At the same time, the development of anti-HMPV vaccines still lag behind RSV, despite the high prevalence of this viral infection in infants.

We previously presented and described Metavac®, a LAV candidate against HMPV, demonstrating strong immunogenicity, protective properties against lethal HMPV challenge in mice, and production scalability for manufacturing purposes^44,45^. Moreover, as the HMPV genome has previously been described for its property to express additional genes of interest (GFP, luciferase, or additional copies of its own genes)^44,48,52,53^, we hypothesized that Metavac® could offer such an advantageous property, as a versatile LAV platform capable of expressing an exogenous RSV-F protein in order to achieve broad protection against human pneumoviruses. To date, several different viruses, mainly belonging to the *Paramyxoviridae* family, such as parainfluenza type 3 virus (PIV3), have been engineered to express surface glycoproteins of RSV or HMPV^54^; however, the use of human pneumoviruses as a vaccine vector or an HMPV/RSV combination has rarely been described.

In this study, we demonstrated that the addition of a supplementary RSV/A2-F coding gene within the Metavac® genome, between the endogenous F and M2 genes, resulted in efficient rescue of the chimeric Metavac®-RSV virus and subsequent incorporation of RSV-F fusion protein into viral particles, despite a significant increase of Metavac® genome length (Fig. 1). Interestingly, deep RNA sequencing and protein expression analysis (Supplementary Figs. S1 and S2) highlighted genome stability after 10-serial cell passages of Metavac®-RSV on LLC-MK2 cells, supporting the robustness of its design. The size of the inserted exogenous cassette could have an impact on the replication of the chimeric virus, as previously described for recombinant PIV3^54,55^, although the limit of exogenous gene incorporation into the Metavac® genome has not been determined yet. Interestingly, we noticed that the position of the RSV-F insertion was critical for the rescue and replication of the recombinant virus. For example, other insertion sites, such as the 3′-proximal positions in the Metavac® genome, resulted in poorly-replicating or non-replicating viruses (data not shown), contrary to Biacchesi and al.^53^. This discrepancy could be likely caused by the intrinsic property of the HMPV A1/C-85473 strain from which Metavac® was generated, and/or a related unbalanced expression of downstream-localized genes, resulting in the impairment of the Metavac® replicative cycle, as it was described for rB/HPIV3^56,57^ or with virus harboring highly fusogenic phenotype^56^ like that of Metavac® virus.

We reported that Metavac®-RSV replicates efficiently in LLC-MK2 cells over a 7-day period, with similar levels to those obtained with Metavac®, while displaying delayed replication kinetics (Fig. 1). Moreover, we observed that Metavac®-RSV induced the formation of large multinucleated cells (Fig. 2). Together with the conserved hyperfusogenic phenotype from the C-85473 parental strain^47,48^, it is possible that the additional and efficient expression of the RSV-F protein also contributes to the membrane fusion mechanism in vitro, thus impacting the propagation properties of the Metavac®-RSV.

We then investigated RSV-F expression by immunofluorescent staining at 48 h post-infection and we observed that half of the LLC-MK2 cells infected by Metavac®-RSV expressed both RSV-F and HMPV-F proteins at their surface (Fig. 2a). This observation correlates with the delayed onset of Metavac®-RSV replication (Fig. 1). Foreseen excision of the GFP gene to reduce Metavac®-RSV genome length and longer expression kinetics studies would be useful to verify if the delay in the onset of replication and protein expression is associated with the increase in the genome length. In a complementary way, co-localization of RSV and HMPV-F proteins, as revealed by immunostaining (Fig. 2a), might also lead to the expression of hypothetical heterologous fusion protein trimers and/or steric shielding of some epitopes and prevention of their recognition by antibodies. Although not intrinsic to all hyperfusogenic proteins, their surface expression is sometimes associated with decreased trafficking of the antigen on the surface, as demonstrated for HMPV and some mutants of a measles virus^48,58^. These could also result in a seemingly low expression level of RSV-F protein on the virion surface, although viral particles embedding both HMPV and RSV-F proteins were visualized in TEM (Fig. 1b).

In the HAE model (Fig. 3) as well as in LLC-MK2 cells (Fig. 1), Metavac®-RSV shows mild-attenuated replicative properties, in comparison to its monovalent Metavac® counterpart. This could be explained by the attenuating effect of the additional gene expression, a phenomenon frequently described in vector vaccines^54^. The putative increase in the fusogenic activity of the Metavac®-RSV virus due to the RSV-F protein expression must be also considered and further investigated^59^. Most importantly, the bivalent candidate was characterized as efficiently infectious and replicative in such a human-differentiated airway epithelial tissue, as expected for an LAV candidate. Notably, the bivalent Metavac®-RSV expressed both RSV-F and HMPV-N proteins in the cilia at the apical surface of HAE (Fig. 3d), where new virions bud from the cell membrane^60^, and also where resident macrophages initiate immune responses^61–63^ and where secreted IgA (sIgA), the main humoral effector, is expressed^64–66^. Conserving replicative properties comparable to what is described of HMPV viruses, Metavac®-RSV proved to be an LAV platform suitable for respiratory epithelium infection.

In line with in vitro results, we reported that Metavac®-RSV also replicated efficiently in vivo, similar to the Metavac® candidate in BALB/c mice (Fig. 4). Importantly, the bivalent Metavac®-RSV replicates in the respiratory tract of infected BALB/c mice as efficiently as the rHMPV, but without virus-associated weight loss, and with reduced lung inflammation and histopathology damage (Fig. 4), as expected for LAV candidates.

Following a double vaccination regimen (prime and boost vaccination by the IN route) with Metavac®-RSV, mice were protected from subsequent rRSV-Luc challenge with a significant reduction of luciferase activity in the upper and lower respiratory tracts of challenged mice, compared to mock-vaccinated animals, and a 10-fold reduction in pulmonary viral titers as measured by RT-qPCR (Fig. 7). Moreover, similar to previous results with the monovalent Metavac®, we demonstrated that mice vaccinated with the bivalent Metavac®-RSV were also completely protected against a lethal HMPV challenge, resulting in a 4–5 log10 decrease in pulmonary viral titers compared to mock-vaccinated animals (Fig. 5). In line with these results, a broad antibody response (NAbs and IgGs) against both RSV and HMPV was detected in sera 20 days after the second immunization with Metavac®-RSV LAV candidate, with a further increase after virus challenge (Figs. 6–7). Interestingly, IgG induced by Metavac®-RSV LAV candidate in mice had a strong affinity to the preF conformation of an RSV F protein, which has been described to be the most efficient neutralizing antibodies^51^. In further studies, it would be interesting to associate such a conformation of RSV-F with our Metavac® LAV platform and investigate the driven humoral responses. Importantly, we also measured the induction of NAbs against heterologous HMPV and contemporary RSV A and RSV B strains (Fig. 6 and Supplementary Fig. S4), demonstrating the potential of the bivalent Metavac®-RSV LAV candidate to confer protection against several RSV and HMPV strains from the two major groups (A and B). Additionally, and similar to the monovalent Metavac®, Metavac®-RSV vaccination was not associated with high immunopathology score and/or an exacerbated immune response in the lungs of challenged mice, in contrast to the group vaccinated by the IM route with the split inactivated HMPV vaccine, suggesting a lower risk for Metavac® and Metavac®-RSV to predispose to EPD.

To our knowledge, our study describes, for the first time, a bivalent HMPV-based LAV candidate that replicates in vitro and in vivo and expresses both HMPV and RSV F antigens. We demonstrate that such a vaccine candidate administered through intranasal instillation induces homologous and heterologous neutralizing antibody responses that contribute to the efficient protection against both RSV and HMPV challenges. Further investigations in complementary (cotton rat) and more relevant preclinical (non-human primate) models must be conducted to confirm our results and to identify efficient dose vaccination strategies. Importantly, the mucosal secretory responses to Metavac®-RSV LAV vaccination in the upper airway epithelium should be characterized in further study, since it has been described that the role of mucosal immunity in controlling respiratory infections was major compared to that of systemic immunity^64,65,67^. Nasal secretory IgAs, which are more cross-protective than other immunoglobulins and initiate antibody-dependent cell-mediated cytotoxicity^68,69^, should be particularly investigated in non-human primate models, as they seem to be the best correlate of protection in challenge studies with RSV^65^ and other respiratory viruses^70,71^.

The development of new vaccines against respiratory mucosal viruses remains a striking challenge, despite strong efforts in this field. In this study, we demonstrated that Metavac® can be used as a versatile LAV platform for heterologous respiratory viral antigen expression. By co-expressing RSV-F and HMPV-F antigens, Metavac®-RSV constitutes an advantageous intranasal LAV candidate, which could confer extended humoral and cellular protections against the two prevalent respiratory pneumoviruses RSV and HMPV, responsible for a major part of bronchiolitis and pneumonia in infants and in the elderly. Associated with a scalable production process for manufacturing, the bivalent Metavac®-RSV LAV candidate could be a new promising option to protect children, at-risk young adults, and the elderly populations that need appropriate specific strategies in terms of vaccine response, schedule, and regimen^72^.

## Methods

### Cells and viruses

LLC-MK2 (ATCC CCL-7) cells were cultivated in minimal essential medium (MEM, Life Technologies) supplemented with 10% fetal bovine serum (FBS, Wisent, St. Bruno, QC, Canada), 1% penicillin/streptomycin (Pen/Strep, 10,000 U/mL, Gibco, ThermoFisher Scientific, Waltham, MA, USA) and 2% L-glutamin (L-Glu, Gibco, ThermoFisher Scientific, Waltham, MA, USA). HEP-2 (ATCC CCL-23) cells were cultivated in MEM medium supplemented with 5% FBS, 1% Pen/Strep, and 2% L-Glu. Vero cells (ATCC CCL-81) were cultivated in MEM medium 4,5 g/l glucose supplemented with 5% FBS, 1% Pen/Strep, and 2% L-Glu. BHK-T7 cells (a kind gift from Dr Ursula Buchholz at the NIAID in Bethesda, MD) were maintained in MEM supplemented with 10% FBS, 1% Pen/Strep, additionally supplemented with 1% non-essential amino acids (NEAA, Life Technologies) and 0.2 mg/mL geneticin (G418, Life Technologies) added every other passage.

Recombinant HMPV viruses rC-85473-GFP (rHMPV), rCAN98-75-GFP, Metavac® (ΔSH-rC-85473-GFP) and Metavac®-RSV were rescued and produced using BHK-T7 and LLC-MK2 cells, as previously described^47^. Recombinant RSVs expressing fluorescent proteins: GFP (rRSV-GFP), mCherry (rRSV-mCh), and Luciferase (rRSV-Luc).

### Molecular biology

RNA of RSV strain A2 virus was isolated from cell culture (Qiamp MiniElute Viral RNA Spin Protocol) and reverse-transcribed with Superscript II RT reverse transcriptase (ThermoFisher Scientific, 18064014). The cDNA product was used as a matrix for amplification of RSV-F ORF using Q5 DNA polymerase (New England BioLabs, M0491L) with appropriate primers (forward: 5′- GAGTGGGACAAGTGAAAATGG-3′, reverse: 5′-GATTTGTCCCAAATTTTTATTTTTATTTTATTTTAATTTTAATTTTATTTTATTTTAATTTAATTTACTTTATTTTTAATTAATTAGTT-3′). RSV-F gene was flanked by HMPV-derived Gene Start and Gene End signals (Fig. 1A), and HMPV genome overlapping regions were added at the 5′ and 3′ extremities of the RSV-F amplicon.

The pSP72 plasmid containing the complete genome of Metavac® (pSP72-ΔSH-rC-85473-GFP/ pSP72-Metavac®) virus^44,45^ was amplified with Q5 DNA polymerase using primers matching the intergenic F-M2 region (forward: 5′- AACTAATTAATTAAAAATAAAGTAAATTAAATTAAAATAAAATAAAATTAAAATTAAAATAAAATAAAAATAAAAATTTGGGACAAATC-3′, and reverse 5′-CCATTTTCACTTGTCCCACTC-3′).

The RSV-F amplicon was then inserted into the linearized pSP72-Metavac® vector by Gibson Assembly® Cloning Kit (New England Biolabs, E5510S) in a 2-fragment cloning reaction, following the provider’s recommendations. Briefly, 75 ng of a linearized vector with a 3-fold molar excess of the insert was used. The reaction product was then diluted 4 times in distilled water, and 2 µl were transformed into Stellar™ Competent Cells (Takara Bio). Bacteria were plated in a selective medium containing Ampicillin and plasmids were isolated by Gene Elute Plasmid Purification Kit (Sigma-Aldrich). The complete plasmid DNA sequence was confirmed by Sanger sequencing.

### Reverse genetics

BHK-T7 cells at 75% confluency were co-transfected with four supporting plasmids encoding ORFs of N, P, L, and M2-1 of HMPV strain B2/CAN98-75, as well as with pSP72 plasmid containing the full-length antigenome of Metavac®-RSV virus using Lipofectamine 2000 (ThermoFisher Scientific, Life Technologies), according to a previously described protocol^47^. Transfected cells were incubated at 37 °C and 5% CO_2_ for 2 days until the GFP expression was noticeable. Next, LLC-MK2 cells were added for co-culture in OptiMEM infection medium supplemented with fresh 0.0002% trypsin, as previously described^47^. Cells were scraped, sonicated, and centrifuged, and the supernatant was diluted to inoculate newly seeded LLC-MK2 monolayers. After several cell passages, recombinant Metavac®-RSV virus was concentrated by ultracentrifugation at 28,000 rpm, resuspended in OptiMEM, and stored at −80 °C. Viral stocks were titrated as 50% tissue culture infectious doses (TCID_50_)/ml.

### Immunostaining

For various immunostaining assays, we used the humanized anti-RSV-F monoclonal antibody (mAb) Palivizumab (Synagis®, AstraZeneca™), anti-HMPV-F mAb (HMPV24, Abcam ab94800), anti-HMPV-N mAb (HMPV123, Abcam ab94803), in-house polyclonal HMPV- or RSV-specific murine sera, respectively generated by mouse infection with HMPV C-85473 or RSV A2 viruses.

For flow cytometry assays, HMPV24 mAb was conjugated with fluorochrome Alexa Fluor™ 647 (Alexa Fluor 647 Antibody Labeling Kit, Invitrogen, A20186), and Palivizumab was conjugated with fluorochrome R-Phycoerythrin (PE/R-Phycoerythrin Conjugation Kit - Lightning-Link®, Abcam, ab102918).

### Transmission electron microscopy

Metavac® and Metavac®-RSV viruses were produced in LLC-MK2 cells and concentrated by ultracentrifugation, as previously described^44^. Viral pellets were then resuspended in 0.9% NaCl and passed through a 0.45 µm filter. Viral suspensions were adsorbed on 200-mesh nickel grids coated with formvar-C for 10 min at room temperature (RT). Immunogold labeling was performed the next day by flotation of the grids on drops of reactive media. Nonspecific sites were coated with 1% BSA in 50 mM Tris-HCl (pH 7.4) for 10 min at RT, then incubated in a wet chamber with Palivizumab diluted in 1% BSA, 50 mM Tris-HCl (pH 7.4) for 2 h at RT. The grids were washed successively in 50 mM Tris-HCl (pH 7.4 and then pH 8.2), incubated with 1% BSA, 50 mM Tris-HCl (pH 8.2) for 10 min at RT, and labeled with 15 nm gold conjugated goat anti-human IgG (Aurion) diluted 1/50 in 1% BSA, 50 Mm Tris-HCl (pH 8.2) for 45 min. A second immunogold labeling with in-house anti-HMPV murine serum was then performed following the same protocol. Finally, the immunocomplex was fixed with 2% glutaraldehyde diluted in 50 mM Tris-HCl (pH 7.4) for 2 min, and grids were stained with UranyLess (Electron Microscopy Sciences, 22409) for 1 min and observed on a TEM (Jeol 1400 JEM, Tokyo, Japan) equipped with a Gatan camera (Orius 1000) and Digital Micrograph Software.

### Replication kinetics

Confluent monolayers of LLC-MK2 cells were washed with PBS and infected with a MOI of 0.01 of Metavac®-RSV or Metavac® vaccine candidates diluted in OptiMEM. Cells were incubated for 1.5 h at 37 °C, then infectious media was aspirated and replaced by fresh OptiMEM with 0.0002% trypsin. Infected cells were incubated at 37 °C and 5% CO_2_ and supernatants were harvested in triplicate at daily intervals for 7 days and then frozen at −80 °C. Each sample was thawed and used for the determination of TCID_50_/ml in LLC-MK2 cells.

### Confocal microscopy

For confocal microscopy observations, confluent monolayers of LLC-MK2 cells grown on Lab-Tek II chamber slides (ThermoFisher Scientific) were infected with a MOI of 0.01 of recombinant Metavac®, Metavac®-RSV or rRSV-GFP viruses. After 3 days of infection, infected cells were fixed with 4% paraformaldehyde in PBS for 30 min at 4 °C, washed in PBS 1X, permeabilized with 0.1% Triton X-100 in PBS (PBS-T), and blocked with 1% SVF for 30 min. Then, anti-RSV-F Palivizumab and anti-HMPV-F HMPV24 antibodies were used as primary antibodies in PBS-T at 1/5000 and 1/500 dilutions, respectively. After 1 h-incubation, the cells were washed in PBS-T and then incubated with goat anti-human mAb conjugated with AlexaFluor 546 and goat anti-mouse mAb conjugated with AlexaFluor 633 (ThermoFisher Scientific) for 30 min at 1/100 dilution. Nuclei were counterstained with DNA-binding fluorochrome 4,6-diamidinon-2-phenylindole (DAPI, Invitrogen). After staining, the coverslips were mounted with Fluoromount G (Cliniscience) and analyzed using a confocal inverted microscope (Zeiss Confocal LSM 880).

### Flow cytometry

For flow cytometry assays, confluent monolayers of LLC-MK2 cells grown in 24-well plates were infected with an MOI of 0.5 of Metavac®-RSV or Metavac® vaccine candidates. After 1.5 h of virus adsorption, the infection medium was replaced by fresh OptiMEM with 0.0002% trypsin. At 48 h post-infection, cells were washed with cold PBS, trypsinized, and resuspended in cold PBS supplemented with 2% FBS. A wash with cold PBS 2% FBS was performed between each step involving antibodies. First, cells were incubated with an optimized concentration of viability dye (LIVE/DEAD™ Fixable Near-IR Dead Cell Stain Kit, ThermoFisher Scientific, L34975) for 30 min at 4 °C. After subsequent washes, samples were incubated for 30 min at 4 °C with optimized concentration of HMPV24 mAb conjugated with Alexa Fluor 647 and Palivizumab conjugated with R-Phycoerythrin. Cells were sorted and analyzed by LSR II Flow Cytometer (BD biosciences®) cytometer to determine: the percentage of infected GFP-positive cells, the percentage of GFP-positive cells with simultaneous HMPV-F and RSV-F expression revealed by HMPV24 antibody, and Palivizumab, respectively. Compensation control for the viability dye was performed with live and dead LLC-MK2 cells. Compensation controls for conjugated antibodies were performed using compensation beads (UltraComp eBeads™ Compensation Beads, ThermoFisher Scientific, 01-2222-42). Approximately 30,000 single live cells were counted per sample, and the experiment was performed in triplicate.

### Infection of reconstituted HAE

In vitro reconstituted HAE, derived from healthy donors’ primary nasal cells (MucilAir™), was purchased from Epithelix (Plan-les-Ouates, Switzerland). HAEs were incubated with a MOI of 0.1 of Metavac® or Metavac®-RSV for 2 h at 37 °C, 5% CO_2_. Infections were monitored for 7 days post-infection (dpi). At 3, 5, and 7 dpi, apical washes with warm OptiMEM were performed in order to extract viral RNA (QIAamp Viral RNA kit, Qiagen, Hilden, Germany), and the images of infected HAEs were taken by fluorescent microscopy with EVOS M5000 Cell Imaging System (Invitrogen, ThermoFisher Scientific).

For fluorescence immunostaining, infected HAEs with a MOI of 0.1 of Metavac®, Metavac®-RSV or rRSV-GFP were rinsed three times with 1X Dulbecco’s PBS (DPBS, Gibco, 14190) at 3 dpi and fixed for 50 min in 4% paraformaldehyde solution (Electron microscopy science, 15710) at RT. HAEs were rinsed three more times in DPBS, then the tissue was embedded in paraffin, and sections of 5 µm-thick slices were prepared using a microtome. Immunostaining was then performed with Discovery XT (Roche) device. Fixed tissues were first deparaffinized and incubated with RiboCC citrate buffer (pH 6.0) for 16 min. The slices were subsequently stained with primary antibodies Palivizumab and HMPV123 mAb at 1:1000 or 1:100 dilutions, respectively, for 1 h at 37 °C, and then with secondary antibodies (Alexa 488 GAR Invitrogen, A11 008 or Alexa 594 GAH Invitrogen™, A11 014) at 1:500 or 1:300 dilution, respectively, for 1 h at 37 °C. The nuclear staining was performed with DAPI. The images were acquired with an inverted confocal microscope (Zeiss Confocal, LSM 880).

### Real-time RT-PCR

The RNA was reverse-transcribed at 42 °C using SuperScript™ II RT (Invitrogen) with random primers. Amplification of the HMPV-N gene was performed by RT-qPCR using Express one-step SYBR GreenER mix, premixed with ROX (ThermoFisher Scientific) and with forward primer 5′-AGAGTCTCAGTACACAATAAAAAGAGATGTGGG-3′ and reverse primer 5′-CCTATTTCTGCAGCATATTTGTAATCAG-3, and amplification of the RSV-F gene was performed using forward primer 5′-CTGTGATAGARTTCCAACAAAAGAACA-3′ and reverse primer 5′-AGTTACACCTGCATTAACACTAAATCC-3′. The calibration of HMPV-N and RSV-F copies was assessed by amplification of a plasmid.

### Animal studies

For in vivo infection studies, 4–6-week-old BALB/c mice (Charles River Laboratories), randomly housed in groups of 5–6 per micro-isolator cage, were infected *via* IN route with 5 × 10^5^ TCID_50_ of rHMPV, Metavac® or Metavac®-RSV, based on previous study^44^, under ketamine/xylazine anesthesia. As a control group, mice were mock-infected IN with OptiMEM medium. Animals were monitored daily for 14 days for weight loss, clinical disease signs, reduced activity, or ruffled fur, and were euthanized upon 20% loss of the initial weight. Mice were euthanized using sodium pentobarbital at 2 dpi (*n* = 2/group) to perform BALs for viral genes quantification by RT-qPCR, or at 5 dpi (*n* = 3/group) to harvest their lungs for histopathological analysis. For histopathological analysis, whole lungs were perfused with 2% formaldehyde at the time of the harvest, embedded in paraffin, and tissue sections were stained with hematoxylin-eosin. Each of the following compartments (interstitium, alveoli/intra-alveolar, peribronchial, perivascular, intrabronchial, and pleural) was scored from 0 (normal) to 4 (severe) based on inflammation criteria (NovaXia Pathology Laboratory). Retrospectively, the quantification of viral gene expression by RT-qPCR was also performed from fixed lung slices after total RNA extraction using RNeasy® DSP FFPE Kit (Qiagen), following manufacturer instructions.

For the vaccination studies, 4–6 week-old BALB/c mice were immunized twice with a 21-day interval before receiving a viral challenge 21 days after the last immunization. Animals were monitored daily for 14 days after each immunization or infection for weight loss, clinical signs, reduced activity, or ruffled fur and were euthanized upon 20% loss of their initial weight.

To assess the protection against the HMPV challenge, sixteen animals were immunized by the IN route with 5 × 10^5^ TCID_50_ of Metavac® or Metavac®-RSV, or by IM route with HMPV split preparation consisting of inactivated HMPV C-85473 virus, as previously described^73^, diluted 1:1 with squalene-based oil-in-water nano-emulsion AddaVax™ (Invivogen). Mice mock-infected IN with OptiMEM (mock vacc.) were used as a negative control vaccination group. Twenty-one days after the second immunization, each mouse was infected with 2 × 10^6^ TCID_50_ of rHMPV, expected to induce lethality in >80% of the animals. At 2 days post-challenge (dpc), mice were euthanized (*n* = 2/group) to collect NW and BALs in PBS 1× to measure HMPV-N gene copies by RT-qPCR. Viral titers (*n* = 4/group) and histopathological scores (*n* = 3/group) were evaluated at 5 dpc from lung homogenates or from formaldehyde-fixed tissues, respectively, as previously described^44^. Prior to immunizations (day −1 or day 20), prior to challenge (day 41), and 21 days after challenge (day 63), blood samples were taken by sub-mandibular bleeding or cardiac puncture at the terminal time-point to evaluate neutralizing antibodies (NAbs) and IgG titers.

To evaluate the protection against the RSV challenge, twelve animals were immunized by the IN route with 5 × 10^5^ TCID_50_ of Metavac®-RSV or with 5 × 10^5^ PFU of rRSV-mCh viruses. As a negative control group of vaccination, mice were mock-infected IN with OptiMEM (mock vacc.). Twenty-one days after the second immunization, each mouse was infected with an inoculum of 3.75 × 10^5^ PFU of rRSV-Luc virus, as previously described^74^. To determine in vivo bioluminescence intensity, mice (*n* = 8/group) were anesthetized 3 and 5 dpc and observed alive using the IVIS imaging system 5 min after IN injection of D-luciferin. At 4 dpc, mice were euthanized (*n* = 4/group), lungs homogenized in 1 ml of PBS 1× before total RNA extraction, and the quantification of RSV-F and HMPV-N genes by RT-qPCR was performed as previously described. Prior to immunizations (days −1 or day 20), prior to challenge (day 41), and 21 days after challenge (day 63), blood samples were taken by sub-mandibular bleeding or cardiac exsanguination at the terminal time-point to evaluate neutralizing antibody (NAb) and IgG titers.

### Neutralization assays

To evaluate the production of a specific neutralizing antibody response, sera were recovered from blood samples, pooled, and heat-inactivated at 56 °C until testing. Serial two-fold dilutions of sera in the infection medium were then tested for neutralization of homologous rHMPV (rC-85473-GFP), heterologous HMPV (rCAN98-75-GFP) or rRSV-mCh viruses on LLC-MK2 cells or Vero cells, respectively. Reciprocal neutralizing antibody titers were determined by an endpoint dilution assay, based on fluorescent detection (Spark® multimode microplate reader, TECAN). Neutralization of infection was defined as >75% decrease in the fluorescence, compared to the negative infection control.

### IgG quantification by ELISA assays

To detect HMPV-, RSV- or preF RSV-specific IgG in mice sera, NUNC Maxi-Sorp 96-well plates (ThermoFisher Scientific) were coated with inactivated virus stocks (HMPV C-85473 or RSV A2 strains, respectively) at 4 µg/ml or recombinant preF RSV protein at 2 µg/ml diluted in carbonate-bicarbonate buffer (0.1 M, pH 9,6).

The PreF RSV protein was obtained by transfection of a pcDNA 3.1+ plasmid encoding DS-Cav1 preF A2 ORF into Expi293F cells using ExpiFectamine 293 reagent (Expi293™ Expression System Kit, ThermoFisher Scientific), as previously described^75^.

Plates were subsequently blocked with 5% milk in PBS-T and incubated with serum samples diluted in 5% milk in PBS-T. Specific IgG antibodies were detected using an anti-mouse IgG-HRP mAb (SouthernBiotech, ref 1031-05). ELISAs were developed using tetramethylbenzidine (TMB SureBlue, SeraCare), and the reaction was stopped with 2 N H_2_SO_4_. Background from empty control wells was subtracted to acquire final absorbance values at 450 nm, and the results were represented as arbitrary units to compare IgG titers at an optimal serum dilution.

### Ethics and biosecurity

HMPV animal studies were approved by the SFR Biosciences Ethics Committee (CECCAPP C015 Rhône-Alpes, protocol ENS_2017_019) according to European ethical guidelines 2010/63/UE on animal experimentation. The protocol of RSV challenge was approved by the Animal Care and Use Committee at “Centre de Recherche de Jouy-en-Josas” (COMETHEA) under relevant institutional authorization (“Ministère de l’éducation nationale, de l’enseignement supérieur et de la recherche”), under authorization number 2015060414241349_v1 (APAFiS#600). All experimental procedures were performed in a Biosafety level 2 facility.

### Statistical analysis

Statistical analyses were performed with GraphPad Prism10 using one-way or two-way ANOVA tests.

### Reporting summary

Further information on research design is available in the Nature Research Reporting Summary linked to this article.

### Supplementary information

Supplementary Information

Supplementary Data 1

Reporting Summary

## Data Availability

All data sets of RNA sequencing supporting the findings of this study are available in Supplementary Figs. Resume table in Supplementary Fig. S1, and complete data sets are Supplementary Data 1.
