# Author Correction: Mucosal bivalent live attenuated vaccine protects against human metapneumovirus and respiratory syncytial virus in mice

**DOI:** 10.1038/s41541-024-00917-w

**Published:** 2024-07-09

**Authors:** Daniela Ogonczyk-Makowska, Pauline Brun, Clémence Vacher, Caroline Chupin, Clément Droillard, Julie Carbonneau, Emilie Laurent, Victoria Dulière, Aurélien Traversier, Olivier Terrier, Thomas Julien, Marie Galloux, Stéphane Paul, Jean-François Eléouët, Julien Fouret, Marie-Eve Hamelin, Andrés Pizzorno, Guy Boivin, Manuel Rosa-Calatrava, Julia Dubois

**Affiliations:** 1 Centre de Recherche en Infectiologie of the Centre Hospitalier Universitaire de Québec and Université Laval, Québec, QC G1V 4G2 Canada; 2 International Research Laboratory RESPIVIR France - Canada, Centre de Recherche en Infectiologie, Faculté de Médecine RTH Laennec, 69008, Lyon, France, Université Claude Bernard Lyon 1, Université de Lyon, INSERM, CNRS, ENS de Lyon, France, Centre Hospitalier Universitaire de Québec - Université Laval, QC G1V 4G2, Québec, Canada; 3 CIRI, Centre International de Recherche en Infectiologie, Team VirPath, INSERM U1111, CNRS UMR 5308, ENS de Lyon, Université Claude Bernard Lyon 1, Lyon, France; 4 Virnext, Faculté de Médecine RTH Laennec, Université Claude Bernard Lyon 1, Université de Lyon, 69008 Lyon, France; 5 Vaxxel, 43 Boulevard du onze novembre 1918, 69100 Villeurbanne, France; 6 Université Paris-Saclay, INRAE, UVSQ, VIM, 78350 Jouy-en-Josas, France; 7 CIRI, Centre International de Recherche en Infectiologie, Team GIMAP, Université Claude Bernard Lyon 1, INSERM U1111, CNRS UMR5308, ENS Lyon, Université Jean Monnet Saint-Etienne, Saint-Etienne, France; 8 Nexomis, Faculté de Médecine RTH Laennec, Université Claude Bernard Lyon 1, Université de Lyon, 69008 Lyon, France

**Keywords:** Virology, Live attenuated vaccines, Viral infection

Correction to: *npj Vaccines* 10.1038/s41541-024-00899-9, published online 19 June 2024

In this article, the authors Guy Boivin, Manuel Rosa-Calatrava and Julia Dubois should have been denoted as jointly supervising authors. The original article has been corrected.

