# Supplementary material for: Mucosal bivalent live attenuated vaccine protects against human metapneumovirus and respiratory syncytial virus in mice
Source: NPJ Vaccines. 2024 Jun 19;9:111. doi: 10.1038/s41541-024-00899-9 (PMC11187144; doi:10.1038/s41541-024-00899-9)
Supplement: Supplementary file 1 — Supplementary Information [file 41541_2024_899_MOESM1_ESM.pdf]

Supplementary Figures _ New mucosal bivalent live-attenuated vaccine is protective against Human Metapneumovirus and Respiratory Syncytial Virus in mice

Daniela Ogonczyk-Makowska[1, 2], Pauline Brun[2, 3, 4], Clémence Vacher[1, 2, 3], Caroline Chupin[2, 3, 5], Clément Droillard[2, 3, 4], Julie Carbonneau[1, 2], Emilie Laurent[2, 3, 4], Victoria Duliere[2, 3, 4], Aurélien Traversier[2, 3, 4], Olivier Terrier[3], Thomas Julien[2, 3, 4], Marie Galloux[6], Stéphane Paul[7], Jean-François Eléouët[6], Julien Fouret[2, 3, 8], Marie-Eve Hamelin[1, 2], Andrés Pizzorno[2, 3, 4], Guy Boivin[1, 2], Manuel Rosa-Calatrava[2, 3, 4] and Julia Dubois[2, 3, 4*]

Supplementary figure 1

A_Genome stability of Metavac® virus up to 10 cellular passages (P10)

Nucleic			Protein		Codon		Amino Acid		Alternative Frequency		
Pos	Ref	Alt	Name	Position	Ref	Alt	Ref	Alt	P4	P8	P10
3071	C	A	M	48	CTC	ATC	L	I	<.03	.06	<.03
3203	A	G		92	ACT	GCT	T	A	<.03	.03	<.03
3335	G	A		136	GGC	AGC	G	S	<.03	.34	.29
3438	A	G		170	GAG	GGG	E	G	.03	<.03	<.03
4156	G	A	F	114	GCA	ACA	A	T	.08	.22	.48
4694	A	G		293	GAA	GGA	E	G	<.03	.08	.58
4703	G	A		296	GGA	GAA	G	E	<.03	.05	<.03
5041	A	G		409	ATA	GTA	I	V	<.03	.04	<.03
6467	A	G	G	63	CAT	CGT	H	R	<.03	<.03	.03
6820	A	G		181	ACA	GCA	T	A	<.03	.03	<.03
9912	A	C	L	904	GAA	GAC	E	D	<.03	.04	<.03
10507	A	C		1103	ATA	CTA	I	L	<.03	.03	<.03
12101	A	G		1634	AAT	AGT	N	S	<.03	.04	.41

B_Genome stability of Metavac®-RSV virus up to 10 cellular passages (P10)

Nucleic			Protein		Codon		Amino Acid		Alternative Frequency			
Pos	Ref	Alt	Name	Position	Ref	Alt	Ref	Alt	P2	P5	P8	P10
2108	A	C	P	32	AAA	AAC	K	N	<.03	.03	<.03	<.03
3108	A	G	M	60	TAT	TGT	Y	C	<.03	.08	.18	.26
3686	A	G		253	AGA	GGA	R	G	<.03	.07	.08	.08
5360	A	G	F	515	AAA	AGA	K	R	<.03	<.03	<.03	.03
6118	C	T	F_RSV	214	TCA	TTA	S	L	<.03	<.03	<.03	.05
7199	A	T		574	TAA	TAT	*	LIKNKVN*	<.03	.10	.28	.31
8104	G	A	G	20	CGT	CAT	R	H	<.03	<.03	.04	.06
8506	G	A		154	AGG	AAG	R	K	<.03	<.03	.03	<.03
9891	A	G	L	309	AAT	GAT	N	D	<.03	.04	.10	.10
11301	G	A		779	GAT	AAT	D	N	<.03	<.03	<.03	.03
12255	C	A		1097	CTA	ATA	L	I	<.03	.05	<.03	<.03
12383	A	T		1139	GAA	GAT	E	D	<.03	<.03	.09	.08
12945	A	G		1327	ACT	GCT	T	A	<.03	.05	<.03	<.03

Supplementary Figure 1. Filtered Mutations Identified in Metavac® and Metavac®-RSV viruses produced after 10 cell passages.

Recombinant viruses Metavac® and Metavac®-RSV were rescued and produced through 10 serial passages in LLC-MK2 cell culture. Viruses were passaged every 7 days after 1 : 10 000 dilution in fresh infection medium. Viral supernatant were harvested at selected passages (P2, P5, P8 and P10). Library preparation was performed from the RNA extracted from the supernatant using the Illumina Stranded Total RNA Prep with Ribo-Zero Plus kit, following the manufacturer's recommendations. The prepared libraries were sequenced on an Illumina NovaSeq platform, using a 2x150 bp paired-end configuration. Raw fastq files were used as input to the viral-recon (Zenodo. https://doi.org/10.5281/zenodo.7764938) nf-core (https://doi.org/10.1038/s41587-020-0439-x) nextflow (https://doi.org/10.1038/nbt.3820) pipeline (v 2.6.0) using default parameters. The resulting alignment file were reprocessed with samtools (v1.9) (https://doi.org/10.1093/gigascience/giab008) and ivar (v1.3.1) (https://doi.org/10.1186/s13059-018-1618-7) to call variants. Variant calling was done only on base with a quality of Q20 minimum. Except the extremity, the coverage was always beyond 1000 for all samples. SNP were called with a minimum frequency of 0.03 and short indel with a minimum frequency of 0.1.

These tables list the viral-coding and non-synonymous mutations found in the (A) Metavac® and (B) Metavac®-RSV vaccine sequence, organized by their position on the reference nucleic acid sequence (denoted as "Nucleic"). For each

mutation, the table provides the reference base ("Ref") and the variant base ("Alt") at that specific nucleotide position. Positions with multiple variant bases are listed in separate rows for clarity. In the "Protein" section, the affected protein or intergenic region is named ("Name"), along with the specific position on the protein. The "Codon" and "Amino Acid" sections detail the sequences for both the reference (Ref) and the variant (Alt), highlighting the changes at coding level. The "Alternative Frequency" columns quantifies the prevalence of the variant sequence among the sequenced reads for each passage.

Complete dataset file is presented in Supplementary Table 1.

Supplementary Figure 2

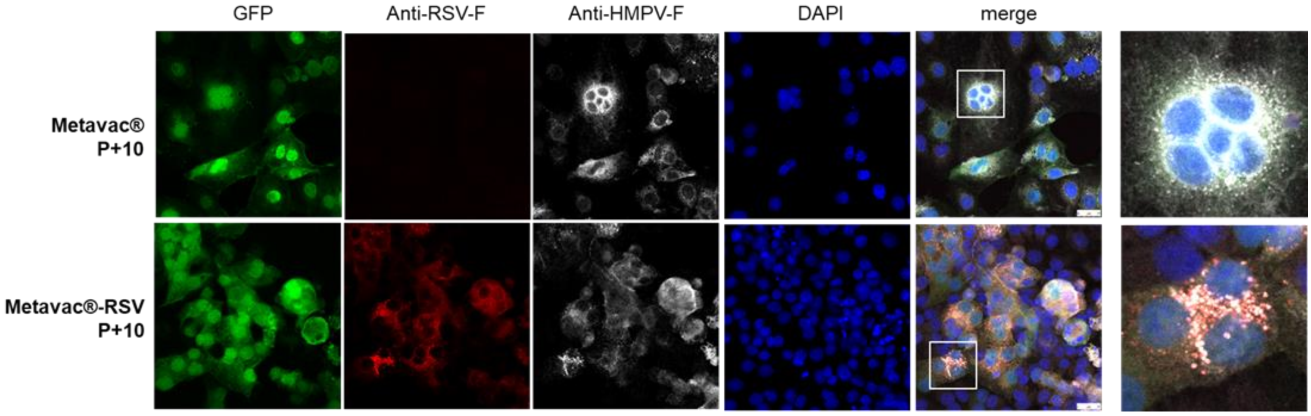

Supplementary Figure 2. Co-immunostaining of HMPV and RSV-F glycoproteins in LLC-MK2 cells infected with P10 virus stocks.

LLC-MK2 cells were infected with GFP-expressing Metavac® and Metavac®-RSV viruses produced after 10 cell passages, fixed and stained at 3 dpi with Palivizumab (red), HMPV24 mAb (white) and DAPI (blue). Merged fluorescent signals are represented (yellow). Images of representative cytopathic effects (CPEs) were taken using Zeiss880 confocal microscope (40x magnification) and processed with ImageJ software. A numeric focus was made on CPEs (square) and presented in the right panel.

Supplementary Figure 3

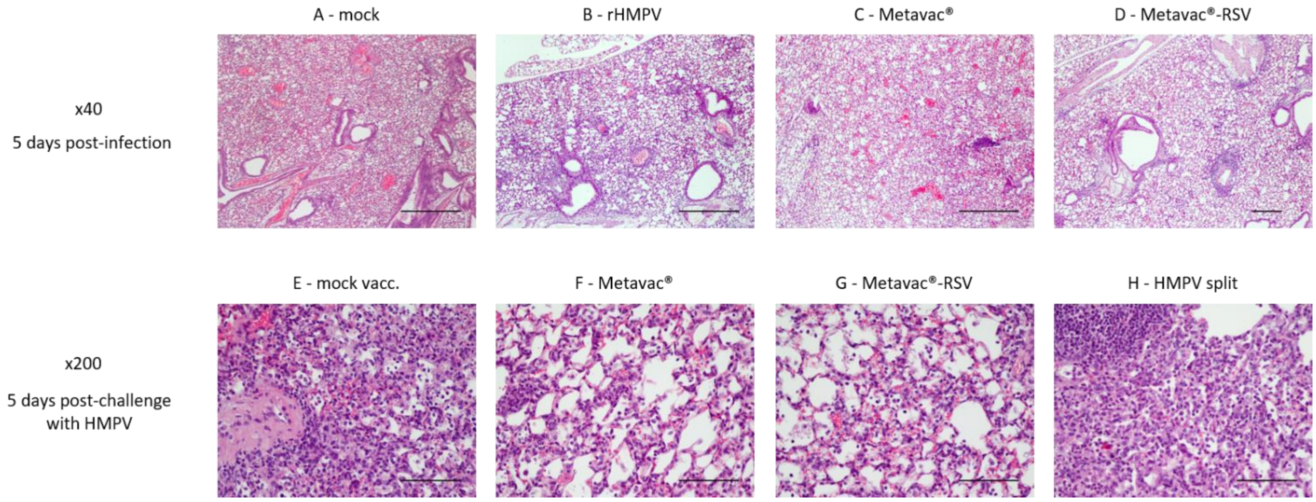

Supplementary Figure 3. Histopathological lung studies from infected (panels A-D) or vaccinated then challenged (panels E-H) mice.

40-fold magnified histopathological images of lung tissues 5 days post-infection: (A) Normal lung parenchyma in a mock-infected animal. (B) Intranasal (IN) infection with rHMPV virus resulted in mild to moderate peri-bronchial and perivascular inflammation. (C) IN infection with monovalent Metavac® LAV candidate resulted in mild interstitial, peri-bronchial, and perivascular inflammation. (D) IN infection with bivalent Metavac®-RSV LAV candidate resulted in mild interstitial, peri-bronchial, and perivascular inflammation. Scale bar = 500µm.

200-fold magnified histopathological images of lung tissues 5 days post-challenge with HMPV: (A) Normal lung parenchyma in a mock-infected animal. (E) Interstitial inflammation with alveolar wall thickening and intra-alveolar inflammatory cells (neutrophils, macrophages) in infected non-vaccinated mice. (F-G) Dual IN immunization with monovalent Metavac® (F) or bivalent Metavac®-RSV (G) vaccine candidates and HMPV challenge resulted in mild (interstitial inflammation). (H) Dual IM immunization with split inactivated HMPV preparation and HMPV challenge resulted in moderate to marked peri-bronchial inflammation, moderate interstitial inflammation, focal pleural inflammation, and marked peri-vascular inflammation. Scale bar = 100µm.

Supplementary Figure 4

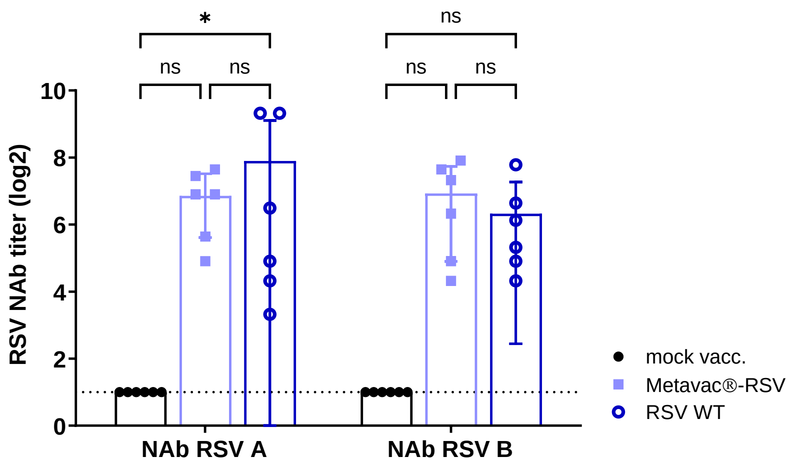

Supplementary Figure 4. Neutralizing antibody response against contemporary RSV A and B strains induced by Metavac®-RSV vaccination. BALB/c mice were immunized twice at a 21-day interval by the IN route with 5x10^5 TCID50 of Metavac®-RSV vaccine candidate or rRSV-mCh (RSV WT) virus. Three weeks after the last immunization, animals (n = 12/group) were inoculated with 1×10^5 PFU of rRSV-Luc virus. Immunogenicity of the Metavac®-RSV LAV candidate was measured by microneutralization assays using two contemporary RSV strains (sequences available at 10.5281/zenodo.10814388) from individual sera collected at 63 dpi, (n = 6). Neutralization was represented as mean log2 reciprocal NAb titer. *, $p < 0.05$, when comparing each group using Two-way ANOVA.
