# Supplementary material for: Mucosal bivalent live attenuated vaccine protects against human metapneumovirus and respiratory syncytial virus in mice
Source: NPJ Vaccines. 2024 Jun 19;9:111. doi: 10.1038/s41541-024-00899-9 (PMC11187144; doi:10.1038/s41541-024-00899-9)
Supplement: Supplementary file 3 — Reporting Summary [file 41541_2024_899_MOESM3_ESM.pdf]

nature portfolio

NPJVACCINES-03525
Corresponding author(s): Julia Dubois
Last updated by author(s): Mar 19, 2024

Reporting Summary

Nature Portfolio wishes to improve the reproducibility of the work that we publish. This form provides structure for consistency and transparency in reporting. For further information on Nature Portfolio policies, see our Editorial Policies and the Editorial Policy Checklist.

Statistics

For all statistical analyses, confirm that the following items are present in the figure legend, table legend, main text, or Methods section.

n/a	Confirmed	
☐	☒	The exact sample size (*n*) for each experimental group/condition, given as a discrete number and unit of measurement
☐	☒	A statement on whether measurements were taken from distinct samples or whether the same sample was measured repeatedly
☐	☒	The statistical test(s) used AND whether they are one- or two-sided<br>*Only common tests should be described solely by name; describe more complex techniques in the Methods section.*
☒	☐	A description of all covariates tested
☒	☐	A description of any assumptions or corrections, such as tests of normality and adjustment for multiple comparisons
☐	☒	A full description of the statistical parameters including central tendency (e.g. means) or other basic estimates (e.g. regression coefficient) AND variation (e.g. standard deviation) or associated estimates of uncertainty (e.g. confidence intervals)
☐	☒	For null hypothesis testing, the test statistic (e.g. *F*, *t*, *r*) with confidence intervals, effect sizes, degrees of freedom and *P* value noted<br>*Give P values as exact values whenever suitable.*
☒	☐	For Bayesian analysis, information on the choice of priors and Markov chain Monte Carlo settings
☒	☐	For hierarchical and complex designs, identification of the appropriate level for tests and full reporting of outcomes
☒	☐	Estimates of effect sizes (e.g. Cohen's *d*, Pearson's *r*), indicating how they were calculated

Our web collection on statistics for biologists contains articles on many of the points above.

Software and code

Policy information about availability of computer code

Data collection	No software used.
Data analysis	Raw fastq files were used as input to the viral-recon (nf-core/viralrecon: nf-core/viralrecon v2.6.0 - Rhodium Raccoon (2.6.0). Zenodo. https://doi.org/10.5281/zenodo.7764938) nf-core (https://doi.org/10.1038/s41587-020-0439-x) nextflow (doi:10.1038/nbt.3820) pipeline (v 2.6.0) using default parameters. The resulting alignment file were reprocessed with samtools (v1.9) (https://doi.org/10.1093/gigascience/giab008) and ivar (v1.3.1) (https://doi.org/10.1186/s13059-018-1618-7) to call variants.

For manuscripts utilizing custom algorithms or software that are central to the research but not yet described in published literature, software must be made available to editors and reviewers. We strongly encourage code deposition in a community repository (e.g. GitHub). See the Nature Portfolio guidelines for submitting code & software for further information.

Data

Policy information about availability of data

All manuscripts must include a data availability statement. This statement should provide the following information, where applicable:

- Accession codes, unique identifiers, or web links for publicly available datasets
- A description of any restrictions on data availability
- For clinical datasets or third party data, please ensure that the statement adheres to our policy

All data sets of RNA sequencing supporting the findings of this study are available in Supplementary Figures. Resume table in Supplementary S1 and the complete

list of variants, including synonymous mutations are available in supplementary dataset Table 1. Moreover, the sequencing raw files are being submitted to ENA (European Nucleotide Archive). The data are accessible under the PRJEB74097 accession number.

Research involving human participants, their data, or biological material

Policy information about studies with human participants or human data. See also policy information about sex, gender (identity/presentation), and sexual orientation and race, ethnicity and racism.

Reporting on sex and gender	*Use the terms sex (biological attribute) and gender (shaped by social and cultural circumstances) carefully in order to avoid confusing both terms. Indicate if findings apply to only one sex or gender; describe whether sex and gender were considered in study design; whether sex and/or gender was determined based on self-reporting or assigned and methods used. Provide in the source data disaggregated sex and gender data, where this information has been collected, and if consent has been obtained for sharing of individual-level data; provide overall numbers in this Reporting Summary. Please state if this information has not been collected. Report sex- and gender-based analyses where performed, justify reasons for lack of sex- and gender-based analysis.*
Reporting on race, ethnicity, or other socially relevant groupings	*Please specify the socially constructed or socially relevant categorization variable(s) used in your manuscript and explain why they were used. Please note that such variables should not be used as proxies for other socially constructed/relevant variables (for example, race or ethnicity should not be used as a proxy for socioeconomic status). Provide clear definitions of the relevant terms used, how they were provided (by the participants/respondents, the researchers, or third parties), and the method(s) used to classify people into the different categories (e.g. self-report, census or administrative data, social media data, etc.) Please provide details about how you controlled for confounding variables in your analyses.*
Population characteristics	*Describe the covariate-relevant population characteristics of the human research participants (e.g. age, genotypic information, past and current diagnosis and treatment categories). If you filled out the behavioural & social sciences study design questions and have nothing to add here, write "See above."*
Recruitment	*Describe how participants were recruited. Outline any potential self-selection bias or other biases that may be present and how these are likely to impact results.*
Ethics oversight	*Identify the organization(s) that approved the study protocol.*

Note that full information on the approval of the study protocol must also be provided in the manuscript.

Field-specific reporting

Please select the one below that is the best fit for your research. If you are not sure, read the appropriate sections before making your selection.

☒ Life sciences ☐ Behavioural & social sciences ☐ Ecological, evolutionary & environmental sciences

For a reference copy of the document with all sections, see nature.com/documents/nr-reporting-summary-flat.pdf

Life sciences study design

All studies must disclose on these points even when the disclosure is negative.

Sample size	We realized iexperimental ndependant triplicate for in vitro experiments.<br>For in vivo experiments, 15-20 mice per groups were used to allow euthanasia of n=2-4 animals per timing for samples analysis, n=6-8 animals for weighing and sera collection.
Data exclusions	No data exclusion.
Replication	Samples generated in our study were systematically aliquoted and frozen for ulterior analyses. Different aliquots of same samples were thawn and resulted in consistent measures. In vivo protocol were reproduced at least 2 times with comparable results. Different manipulators performed analytical assays to avoid manipulator bias. Any results of this study could not be reproduced.
Randomization	Mice were housed randomly in groups of 5-6 per micro-isolator cage. Cages were randomly attributed to experimental groups.
Blinding	External histopathology analysis were blinded.

Reporting for specific materials, systems and methods

We require information from authors about some types of materials, experimental systems and methods used in many studies. Here, indicate whether each material, system or method listed is relevant to your study. If you are not sure if a list item applies to your research, read the appropriate section before selecting a response.

Materials & experimental systems

n/a	Involved in the study	
☐	☒	Antibodies
☐	☒	Eukaryotic cell lines
☒	☐	Palaeontology and archaeology
☐	☒	Animals and other organisms
☒	☐	Clinical data
☒	☐	Dual use research of concern
☒	☐	Plants

Methods

n/a	Involved in the study	
☒	☐	ChIP-seq
☐	☒	Flow cytometry
☒	☐	MRI-based neuroimaging

Antibodies

Antibodies used: anti-RSV mAb Palivizumab (Synagis®, AstraZeneca™); anti-HMPV-F mAb (HMPV24, Abcam ab94800); anti-HMPV-N mAb (HMPV123, Abcam ab94803); in-house polyclonal HMPV- or RSV-specific murine sera

Validation: Validation of Abcam ab94800 and Abcam ab94803 was documented by the provider. Validation of Palivizumab and in-house murine sera for the purpose of this study was made using negative and positive control conditions.

Eukaryotic cell lines

Policy information about cell lines and Sex and Gender in Research

Cell line source(s): Cell lines were provided by ATCC (LLC-MK2 and HEP-2) or by Dr. Buchholz lab (BHK-T7)

Authentication: LLC-MK2 (ATCC CCL-7); HEP-2 (ATCC CCL-23)

Mycoplasma contamination: All cell lines were tested negative for mycoplasma contamination.

Commonly misidentified lines (See ICLAC register): *Name any commonly misidentified cell lines used in the study and provide a rationale for their use.*

Animals and other research organisms

Policy information about studies involving animals; ARRIVE guidelines recommended for reporting animal research, and Sex and Gender in Research

Laboratory animals: BALB/C 5-weeks old Female

Wild animals: The study did not involved wild animals.

Reporting on sex: This information has not been collected

Field-collected samples: The study did not involved sample collected from the field.

Ethics oversight: Study protocols have been approved by SFR Biosciences Ethics Committee (CECCAPP C015 Rhône-Alpes) and Animal Care and Use Committee (COMETHEA, Jouy-en-Josas), under relevant institutional authorization and according to European ethical guidelines 2010/63/UE on animal experimentation.

Note that full information on the approval of the study protocol must also be provided in the manuscript.

Plants

Seed stocks: *Report on the source of all seed stocks or other plant material used. If applicable, state the seed stock centre and catalogue number. If plant specimens were collected from the field, describe the collection location, date and sampling procedures.*

Novel plant genotypes: *Describe the methods by which all novel plant genotypes were produced. This includes those generated by transgenic approaches, gene editing, chemical/radiation-based mutagenesis and hybridization. For transgenic lines, describe the transformation method, the number of independent lines analyzed and the generation upon which experiments were performed. For gene-edited lines, describe the editor used, the endogenous sequence targeted for editing, the targeting guide RNA sequence (if applicable) and how the editor was applied.*

Authentication: *Describe any authentication procedures for each seed stock used or novel genotype generated. Describe any experiments used to assess the effect of a mutation and, where applicable, how potential secondary effects (e.g. second site T-DNA insertions, mosiacism, off-target gene editing) were examined.*

Flow Cytometry

Plots

Confirm that:

☒ The axis labels state the marker and fluorochrome used (e.g. CD4-FITC).

☒ The axis scales are clearly visible. Include numbers along axes only for bottom left plot of group (a 'group' is an analysis of identical markers).

☒ All plots are contour plots with outliers or pseudocolor plots.

☒ A numerical value for number of cells or percentage (with statistics) is provided.

Methodology

Sample preparation	Infected LLC-MK2 (ATCC CCL-7) cells culture were used. Cells were washed with cold PBS, trypsinized, and resuspended in cold PBS supplemented with 2% FBS. A wash with cold PBS 2% FBS was performed between each step involving antibodies. Cells were incubated with an optimized concentration of viability dye (LIVE/DEAD™ Fixable Near-IR Dead Cell Stain Kit, ThermoFisher Scientific, L34975) for 30 min at 4°C. After subsequent washes, samples were incubated for 30 min at 4°C with optimized concentration of HMPV24 mAb conjugated with Alexa Fluor 647 and Palivizumab conjugated with R-Phycoerythrin (PE). Compensation controls for conjugated antibodies were performed using compensation beads (UltraComp eBeads™ Compensation Beads, ThermoFisher Scientific, 01-2222-42).
Instrument	LSR II Flow Cytometer (BD biosciences®)
Software	BD FACSDiva
Cell population abundance	Approximately 30,000 single live cells were counted per each sample performed in triplicate. As LLC-MK2 cell line is used, no mixed cell population is detected.
Gating strategy	Gating of sub-populations were done hierarchically oas : all cells in the sample; single cells; live cells; positive or negative for GFP expression ; AlexaFluor647 positive cells and PE positive cells.

☒ Tick this box to confirm that a figure exemplifying the gating strategy is provided in the Supplementary Information.
